# Greater preservation of SARS‐CoV‐2 neutralising antibody responses following the ChAdOx1‐S (AZD1222) vaccine compared with mRNA vaccines in haematopoietic cell transplant recipients

**DOI:** 10.1111/bjh.19874

**Published:** 2024-11-17

**Authors:** Hayley Colton, Natalie Barratt, Nigel Temperton, Hailey Hornsby, Adrienn Angyal, Irina Grouneva, Benjamin B. Lindsey, Pamela Kearns, Eleanor Barnes, Carl S. Goodyear, Alex Richter, David Thomas, Gordon Cook, Iain B. McInnes, Michelle Willicombe, Stefan Siebert, Kim Orchard, Rachael Selby, Sarah Bowden, Paul J. Collini, Ann Pope, Amanda Kirkham, Barbara Kronsteiner, Susanna J. Dunachie, Paul Miller, Jennifer Clay, Erin Hurst, Ram Malladi, Murali Kesavan, Francesca Kinsella, Robin Sanderson, Kwee L. Yong, Daniel Rea, Helen Parry, PITCH Consortium, OCTAVE Collaborative Group, OCTAVE‐DUO Investigators, PROSECO Investigators, Sean H. Lim, John A. Snowden, Thushan I. de Silva

**Affiliations:** ^1^ Division of Clinical Medicine, School of Medicine and Population Health The University of Sheffield Sheffield UK; ^2^ NIHR Sheffield Biomedical Research Centre and the Florey Institute of Infection University of Sheffield Sheffield UK; ^3^ Viral Pseudotype Unit, Medway School of Pharmacy Universities of Kent and Greenwich Chatham UK; ^4^ Cancer Research UK Clinical Trials Unit (CRCTU) University of Birmingham Birmingham UK; ^5^ Peter Medawar Building for Pathogen Research, Nuffield Department of Clinical Medicine University of Oxford Oxford UK; ^6^ College of Medical, Veterinary & Life Sciences University of Glasgow Glasgow UK; ^7^ Clinical Immunology Service University of Birmingham Birmingham UK; ^8^ The Cambridge Institute for Therapeutic Immunology and Infectious Disease (CITIID) University of Cambridge Cambridge UK; ^9^ National Institute for Health Research, Leeds MIC University of Leeds Leeds UK; ^10^ Department of Immunology and Inflammation, Centre for Inflammatory Disease Imperial College London London UK; ^11^ Department of Haematology University Hospital Southampton NHS Foundation Trust Southampton UK; ^12^ Sheffield Teaching Hospitals NHS Foundation Trust Royal Hallamshire Hospital Sheffield UK; ^13^ British Society of Blood and Marrow Transplantation and Cellular Therapy Guy's Hospital London UK; ^14^ Department of Haematology St James's University Hospital Leeds UK; ^15^ Northern Centre for Cancer Care Freeman Hospital Newcastle UK; ^16^ Department of Haematology Cambridge University Hospitals NHS Foundation Trust Cambridge UK; ^17^ Department of Oncology, Cancer and Haematology Centre Churchill Hospital Oxford UK; ^18^ Centre for Clinical Haematology University Hospitals Birmingham NHS Foundation Trust Birmingham UK; ^19^ King's College Hospital NHS Foundation Trust London UK; ^20^ Department of Haematology, Cancer Institute University College London London UK; ^21^ Centre for Cancer Immunology University of Southampton Southampton UK

**Keywords:** SARS‐CoV‐2, stem cell transplant, vaccination

## Abstract

Whilst SARS‐CoV‐2 mRNA vaccines generate high neutralising antibodies (nAb) in most individuals, haematopoietic stem cell transplant (HSCT) and chimeric antigen receptor T‐cell (CAR‐T) recipients respond poorly. HSCT/CAR‐T treatment ablates existing immune memory, with recipients requiring revaccination analogous to being vaccine naive. An optimal revaccination strategy for this cohort has not been defined. Factors predicting immunogenicity following three ancestral SARS‐CoV‐2 vaccines were assessed in 198 HSCT/CAR‐T recipients and 96 healthcare workers (HCWs) recruited to multicentre studies. Only 25% of HSCT/CAR‐T recipients generated nAbs following one dose, with titres 167‐fold and 7‐fold lower than that in HCWs after the first and second doses, respectively. Lower post‐second dose nAb titres were associated with older age, rituximab use, and previous HSCT. ChAdOx1‐S recipients were more likely to generate nAbs compared with mRNA vaccines, with titres comparable to HCWs. In contrast, nAbs were significantly lower in HSCT/CAR‐T recipients than HCWs after mRNA vaccination. The poor first‐dose immunogenicity in HSCT/CAR‐T recipients suggests a minimum licensed dosing interval could limit the period of vulnerability following HSCT/CAR‐T. The relative preservation of nAbs with ChAdOx1‐S vaccination highlights the importance of evaluating alternative platforms to mRNA vaccination within this highly vulnerable clinical cohort.

## Graphical abstract

Knowledge of factors impacting immune responses after COVID‐19 vaccination is essential for guiding revaccination strategies in haematopoietic stem cell transplant (HSCT) and chimeric antigen receptor T cells (CAR‐T) recipients. Neutralising antibodies (nAbs) were assessed after three vaccine doses, and compared with healthcare workers (HCWs). Immunogenicity was particularly poor following one dose, with only 25% generating nAbs. Following two doses, recipients of ChAdOx1‐S were eight times more likely to generate nAbs than recipients of mRNA vaccines, with titres similar to HCWs. Lower nAb titres were associated with older age, rituximab, and prior HSCT. Using the minimum licensed dosing intervals and exploring other vaccine platforms could benefit this vulnerable group.

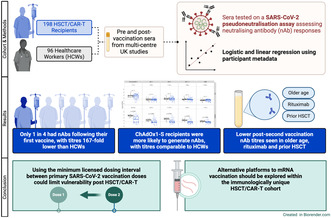

## INTRODUCTION

Recipients of haematopoietic stem cell transplant (HSCT) and chimeric antigen receptor T cells (CAR‐T) continue to be at the highest risk of severe COVID‐19 in the SARS‐CoV‐2 Omicron era.^1^, ^2^, ^3^ Despite a three‐dose primary SARS‐CoV‐2 immunisation course, HSCT‐recipients had a higher incidence of both COVID‐19‐related hospitalisation (11‐fold) and death (5‐fold) compared with the general population in England.^1^ Unlike other immunosuppressed groups, new HSCT/CAR‐T recipients are considered ‘never vaccinated’ as their immune memory is ablated during treatment, so require a repeat primary vaccine course regardless of prior vaccine status.^4^ Therefore, further optimisation of primary SARS‐CoV‐2 immunisation courses with new regimens and vaccine platforms is vital for these patients.

The three‐dose primary SARS‐CoV‐2 vaccination schedule for unvaccinated clinically vulnerable adults in the United Kingdom (UK) was simplified to a two‐dose course in 2023, with the rationale that many individuals had a degree of immune memory from prior infections.^4^, ^5^ A longer interval between first and second doses is also being increasingly adopted, based on data from healthy individuals demonstrating greater antibody responses with extended intervals.^6^ Whether these strategies are appropriate in HSCT/CAR‐T recipients is unclear. The multicentre studies OCTAVE, OCTAVE‐DUO, and PROSECO demonstrated that many immunosuppressed groups were less likely to develop detectable SARS‐CoV‐2 binding IgG responses following two vaccine doses, although some non‐responders seroconverted after a third dose.^7^, ^8^, ^9^

To what degree vaccine type drives SARS‐CoV‐2 vaccine immunogenicity in HSCT/CAR‐T recipients remains relatively under‐explored. In many countries, only mRNA vaccines are recommended for revaccination schedules,^5^, ^10^, ^11^, ^12^ and have been shown to generate higher humoral responses than other vaccines in many populations, including the OCTAVE cohort.^7^, ^13^, ^14^, ^15^, ^16^, ^17^ Knowledge of which factors impact serum neutralising activity after each vaccine dose is important in guiding revaccination strategies in HSCT/CAR‐T recipients. We report on the induction of neutralising antibody (nAb) and cellular immunity following the adenoviral vector vaccine ChAdOx1‐S (AZD1222; AZ) or mRNA vaccine (BNT162b2 or mRNA‐1273) in planned subcohort analyses of HSCT/CAR‐T recipients recruited to several UK multicentre studies of immune‐vulnerable patients.

## MATERIALS AND METHODS

### Study cohorts

OCTAVE (ISRCTN12821688), OCTAVE‐DUO (ISRCTN15354495), and PROSECO (NCT04858568) were prospective multicentre trials in the UK evaluating humoral and cellular responses against SARS‐CoV‐2 in clinically vulnerable adults.^7^, ^8^, ^9^, ^18^ Immune responses following first (V1) and second (V2) vaccinations were studied in OCTAVE, which recruited clinically vulnerable adults between 19 February 2021 and 01 October 2021.^7^ Participants were vaccinated through the National Health Service (NHS) roll out with either mRNA or ChAdOx‐S vaccinations encoding ancestral SARS‐CoV‐2 spike.^7^ Immunogenicity following a third dose (V3) was studied in OCTAVE‐DUO and PROSECO. OCTAVE‐DUO randomised clinically vulnerable adults who failed to mount an adequate binding antibody response after two doses of SARS‐CoV‐2 vaccines to a third dose (primarily BNT162b2 or mRNA‐1273) between 04 August 2021 and 31 March 2022.^9^ PROSECO recruited people with lymphoid malignancies between 11 January 2021 and 07 May 2021, of which a subset were HSCT‐recipients vaccinated via the NHS rollout.^8^, ^19^ All reported humoral immunity data from HSCT/CAR‐T recipients in these cohorts to date were for binding antibody responses.^7^, ^8^, ^9^, ^19^

Healthy participant samples were from PITCH (ISRCTN11041050), a multicentre prospective cohort study of UK healthcare workers (HCWs) evaluating humoral and cellular responses against SARS‐CoV‐2, that began recruiting from 9 December 2020.^18^ Additional prevaccination samples from SARS‐CoV‐2 infection‐naive HCWs from the HERO study were also included.^20^

### Serum selection

All available sera taken within defined post‐vaccine visit windows, with matched metadata, were used from adult HSCT/CAR‐T recipients participating in OCTAVE (21–42 days post‐V1 and post‐V2) and OCTAVE‐DUO (21–35 days post‐V3). As OCTAVE‐DUO only recruited HSCT‐recipients with no (<0.8 AU/mL) or low (0.8–399 AU/mL) receptor binding domain (RBD) antibody responses after two doses,^9^ post‐V3 plasma from HSCT‐recipients participating in PROSECO with anti‐Spike IgG responses >300 BAU/mL after two vaccine doses were included for a ‘normal‐responder group’.^8^ Available post‐vaccination sera from PITCH participants recruited to Sheffield and Oxford sites were selected to correspond with sampling windows in OCTAVE and OCTAVE‐DUO. Prevaccination samples from PITCH and HERO participants were used to characterise nAb in seronegative donors.

### Pseudotyped virus neutralisation assay

NAb responses to ancestral (B.1) and Omicron (BA.1, BA.5, BQ.1.1, XBB) SARS‐CoV‐2 variants were assessed using a pseudotyped virus neutralisation assay. Pseudotyped viruses were produced by co‐transfecting stocks of human embryonic kidney 293 T (HEK‐293 T) cells with plasmids encoding Spike variants, a p8.91‐lentiviral vector, and a firefly luciferase reporter plasmid (pCSFLW) as previously described.^21^ Chinese hamster ovarian cells expressing TMPRSS2 and ACE2 (CHO‐ACE2‐TMPRSS) were used as target cells in nAb assays and were maintained in Ham's F‐12 Nutrient Mix (Gibco 21765029) supplemented with 10% fetal bovine serum (Cytiva SV30160.03) and 1% penicillin–streptomycin (Sigma‐Aldrich P4333).

Sera were diluted 1:20 and mixed 1:1 with pseudotyped viruses (pre‐titrated concentration to achieve 5 × 10^6^ relative lights units; RLU) in white Nunc™ MicroWell™ 96‐Well plates (Thermo Fisher Scientific Inc.) in duplicate, resulting in an initial nAb screening serum dilution of 1:40. Following a 1‐h incubation at 37°C and 5% CO_2_, 20 000 CHO‐ACE2‐TMPRSS cells were added to wells prior to a further 48 h incubation. Supernatant was removed and 30 μL of Bright‐Glo™Luciferase Assay System (Promega E2650) diluted 1:1 with phosphate buffered saline was added to each well. Luminescence in RLU was read on a GloMax® Explorer Microplate Reader (Promega) after 5 min. All samples resulting in a mean RLU ≤50% of the pseudotype virus (no serum) control condition were repeated at serial serum dilutions of 1:40–1:78 732 in duplicate to estimate a half‐maximal neutralising titre (NT50). Each plate included negative (anti‐Spike IgG negative) and positive (pooled SARS‐CoV‐2 convalescent) serum controls.

### Binding antibody and cellular responses

RBD and nucleocapsid binding antibody responses were generated previously in OCTAVE and OCTAVE‐DUO (Elecsys AntiSARS‐CoV‐2, Roche) and PITCH (V‐PLEX COVID‐19 Coronavirus Panel 3, Meso Scale Discovery),^7^, ^18^ whilst anti‐Spike IgG was measured during PROSECO (Meso Scale Discovery).^19^ T‐cell responses in OCTAVE were assessed using the T‐SPOT DISCOVERY SARS‐CoV‐2 assay (Oxford Immunotec) as previously described in a subset of participants.^7^

### Statistical analysis

Sera with an NT50 < 40 were considered non‐neutralising based on non‐specific inhibition seen in anti‐Spike IgG negative sera from SARS‐CoV‐2‐naive HCWs and assigned a random NT50 < 40. Statistical analysis was performed using R version 4.3.1. Comparisons of NT50 across groups were performed using the Mann–Whitney U test, with Bonferroni correction for multiple testing. Differences between groups in the presence of neutralising activity was compared using Fisher's exact test. Correlations were performed using Spearman correlation. Logistic regression was performed to explore covariates predicting the presence of binding or nAb responses after two doses. Linear regression was performed to explore covariates predicting binding antibody or nAb (NT50) titre following two doses. In both cases, variables that either had a *p*‐value of <0.1 on univariate analysis or an a priori hypothesis of impacting vaccine responses were taken forward to multivariable regression. NT50 were log10 transformed for visualisation and statistical analysis. Fold changes in nAb between groups were calculated using absolute NT50 values to ease interpretation.

### Ethical approval

OCTAVE was approved by the UK Medicines and Healthcare Products Regulatory Agency (MHRA) on 5 February 2021 and by the London and Chelsea Research Ethics Committee (REC, 21/HRA/0489) on 12 February 2021. OCTAVE‐DUO was approved by the MHRA on 19 July 2021. PITCH participants in Oxford were recruited under the GI Biobank Study 16/YH/0247, approved by the Yorkshire & The Humber Sheffield REC on 29 July 2016, which has been amended for this purpose on 8 June 2020. In Sheffield, PITCH participants were recruited under the Observational Biobanking study STHObs (18/YH/0441), which was amended for this study on 10 September 2020. PROSECO and HERO were approved by the UK Health Research Authority (IRAS IDs 283461 and 294739/233768). Written informed consent was obtained for all participants enrolled in the above studies. All studies were conducted according to the principles of the Declaration of Helsinki (2008) and the International Conference on Harmonisation Good Clinical Practice guidelines.

## RESULTS

### Participant information

Data from 198 HSCT/CAR‐T recipients recruited to OCTAVE, OCTAVE‐DUO, and PROSECO are included in this paper (Table 1). Fourteen individuals were in both OCTAVE and OCTAVE‐DUO studies. HSCT/CAR‐T recipients were predominantly male (116, 58.6%), of white ethnicity (174, 87.9%) and aged under 65 years (150, 75.8%; median 55 years). Most had undergone allogeneic HSCT (145, 73.2%), with fewer receiving an autologous HSCT (43, 21.7%) or CAR‐T therapy (8, 4.0%). From OCTAVE, sera were available for nAb assays from 72/82 post‐V1 visits and 138/179 post‐V2 visits.^7^ Previously generated post‐V2 RBD IgG data (133) and T‐SPOT data (44) from HSCT/CAR‐T recipients were also analysed.^7^ From OCTAVE‐DUO, 33 HSCT‐recipients had post‐V3 sera available for use in nAb assays.^9^ Post‐V3 plasma from an additional five HSCT‐recipients were available from PROSECO.^8^

**TABLE 1:** Details of participants included from the HSCT/CAR‐T recipients in OCTAVE, OCTAVE‐DUO, and PROSECO and healthy controls from PITCH. The number of participants with available data from post‐V1, post‐V2, and post‐V3 visits are detailed separately.

	HSCT/CAR‐T recipients^a^		Healthy controls
	Post‐V1 and V2	Post‐V3	
Participants who had post‐vaccination sera available, *n*	174 (OCTAVE)	33 (OCTAVE‐DUO) 5 (PROSECO)	96 (PITCH)
Age, years, median (range)	55 (19–75)	61 (33–72)	43 (21–70)
Sex, *n* (%)			
Male	100 (57.5)	23 (60.5)	21 (21.9)
Female	74 (42.5)	15 (39.5)	75 (78.1)
Ethnicity, *n* (%)			
White	156 (89.7)	32 (84.2)	60 (62.5)
Asian	6 (3.4)	1 (2.6)	3 (3.1)
Black	2 (1.1)	0 (0.0)	0 (0.0)
Mixed ethnicities	0 (0.0)	0 (0.0)	2 (2.1)
Other	0 (0.0)	0 (0.0)	1 (1.0)
Unknown	10 (5.7)	5 (13.2)	30 (31.3)
Vaccination type (V1 and V2),^b^ *n* (%)			
mRNA/mRNA	76 (43.7)	14 (36.8)	42 (43.8)
ChAdOx1‐S/ChAdOx1‐S	93 (53.4)	24 (63.2)	54 (56.3)
ChAdOx1‐S/mRNA	3 (1.7)	0 (0.0)	0 (0.0)
Unknown	2 (1.1)	0 (0.0)	0 (0.0)
Dosing interval between V1 and V2, days, median (range)	76 (19–421)	77 (29–188)	63 (34–84)
Vaccination type (V3),^b^ *n* (%)			
mRNA	NA	38 (100.0)	70^c^ (98.6)
ChAdOx1‐S	NA	0 (0.0)	1^d^ (1.4)
Evidence of COVID‐19 prior to post‐V2 visit,^e^ *n* (%)			
Yes	21 (12.1)	1 (2.6)	42 (43.8)
No	117 (67.2)	37 (97.4)	49 (51.0)
Unknown	36 (20.7)	0 (0.0)	5 (5.2)
HSCT‐type, *n* (%)			
Allogeneic	125 (71.8)	30 (78.9)	NA
Autologous	40 (23.0)	7 (18.4)	
CAR‐T	8 (4.6)	0 (0.0)	
Unknown	1 (0.1)	1 (2.6)	
Time between D0 and V1, days, median (range)	241 (20–8278)	379 (106–8287)	NA
Indication, *n* (%)			
Myeloid	116 (66.7)	20 (52.6)	NA
Lymphoid	46 (26.4)	16 (42.1)	
Neurological^f^	6 (3.4)	0 (0.0)	
Other^g^	6 (3.4)	2 (5.3)	
Disease status, *n* (%)			
Complete remission	121 (69.5)	29 (76.3)	NA
Stable disease	10 (5.7)	1 (2.6)	
Partial remission	13 (7.5)	2 (5.3)	
Progressive/relapsed disease	7 (4.0)	2 (5.3)	
Not applicable (neurological)	6 (3.4)	0 (0.0)	
Unknown	17 (9.8)	4 (10.5)	
Conditioning,^h^ *n* (%)			NA
Reduced intensity	73 (42.0)	17 (44.7)	
Myeloablative	25 (14.7)	6 (15.8)	
Non‐myeloablative	12 (6.9)	1 (2.6)	
Unknown/not applicable	64 (36.8)	14 (36.8)	
Lymphocyte depletion,^h^ *n* (%)			NA
Campath	56 (32.2)	18 (47.4)	
Anti‐thymocyte globulin	29 (16.7)	7 (18.4)	
Post‐transplant cyclophosphamide	13 (7.5)	2 (5.3)	
None	10 (5.7)	3 (7.9)	
Unknown/not applicable	66 (37.9)	8 (21.1)	
Other treatments received, *n* (%)			NA
Total body irradiation	29 (16.7)	6 (15.8)	
Previous HSCT	36 (20.7)	3 (7.9)	
Rituximab	19 (10.9)	4 (10.5)	
Tocilizumab	6 (3.4)	0 (0.0)	
GVHD			NA
Acute at V2	7 (4.0)	Data not available	
Chronic at V2	15 (8.6)		
Post‐V1 results available		NA	
RBD IgG	81 (46.6)		0 (0.0)
NAb	72 (41.3)		51 (53.1)
T‐SPOT	0 (0.0)		0 (0.0)
Post‐V2 results available		NA	
RBD IgG	133 (76.4)		0 (0.0)
NAb	138 (79.3)		89 (92.7)
T‐SPOT	44 (25.3)		0 (0.0)
Post‐V3 results available	NA		
RBD IgG		0 (0.0)	0 (0.0)
NAb		38 (100.0)	71 (74.0)
T‐SPOT		0 (0.0)	0 (0.0)

Abbreviations: CAR‐T, chimeric antigen receptor T cells; D0, day zero of HSCT/CAR‐T; GVHD, Graft‐versus‐host disease; HSCT, haematopoetic stem cell transplant; NA, not applicable; nAb, neutralising antibodies; RBD, receptor binding domain; V1, first vaccination; V2, second vaccination; V3, third vaccination.

^a^ Fourteen participants in OCTAVE were recruited into OCTAVE‐DUO.

^b^ mRNA includes BNT162b2 (BioNTech Pfizer) and mRNA‐1273 (Moderna).

^c^ Regimens for V1‐V2: mRNA *n* = 41, ChAdOx1‐S *n* = 29.

^d^ Regimens for V1‐V2: ChAdOx1‐S *n* = 1.

^e^ Defined as previous PCR positivity or detection of SARS‐CoV‐2 anti‐nucleocapsid IgG from Roche Elecsys (OCTAVE and OCTAVE‐DUO) or Meso Scale Discovery (PROSECO and PITCH).

^f^ Neurological conditions include neuromyelitis optica, multiple sclerosis, chronic inflammatory demyelinating polyneuropathy, myasthenia gravis, and stiff person syndrome.

^g^ Other conditions: aplastic anaemia *n* = 5, amyloidosis *n* = 1.

^h^ Data for conditioning and lymphocyte depletion is only available for allogeneic HSCT recipients in OCTAVE and OCTAVE‐DUO.

**FIGURE 1:**
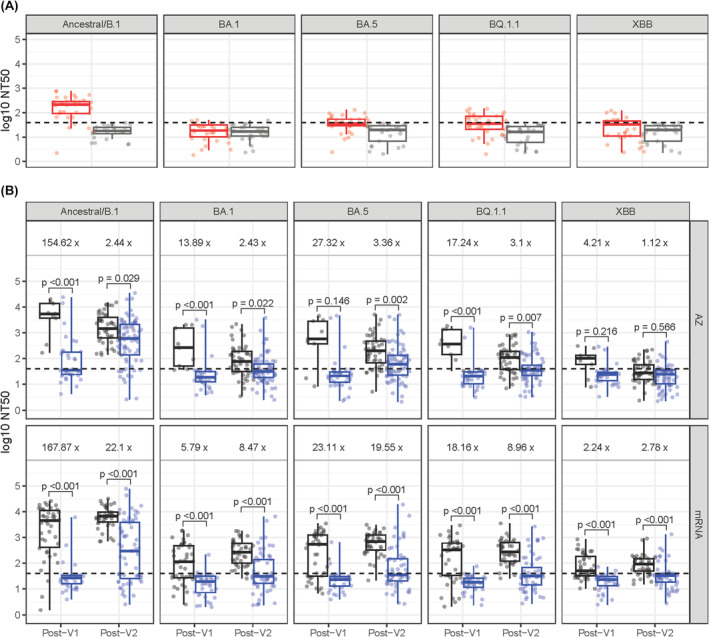
Neutralising antibody titres against SARS‐CoV‐2 ancestral and Omicron variants in HSCT/CAR‐T recipients and healthy controls. (A) Data in healthy controls prevaccination stratified by SARS‐CoV‐2 naive and convalescent serostatus. (B) Data comparing HSCT/CAR‐T recipients with healthy control participants following first and second doses, stratified by receipt of ChAdOx1‐S (AZD1222; AZ) or mRNA vaccines (BNT162b2 or mRNA‐1273). *Y*‐axes denote log10 reciprocal dilution at which 50% neutralisation was observed (NT50). Boxplots demonstrate median and interquartile ranges (IQR25 and IQR75), with whiskers showing the range (Mann–Whitney *U* test and Bonferroni correction for multiple comparisons). Dashed line shows an NT50 of 40, considered the threshold at which true neutralising activity in serum is detected. CAR‐T, chimeric antigen receptor T cell; HSCT, haematopoietic stem cell transplant; V1, first vaccination; V2, second vaccination. [Colour figure can be viewed at wileyonlinelibrary.com]

Samples from 96 HCWs from PITCH were included, comprising 51 post‐V1, 89 post‐V2, and 71 post‐V3 sera (Table 1). Healthy controls were predominantly female (75, 78.1%), with a median age of 43 years. Preimmunisation control sera (59) from PITCH and HERO HCWs were also used (Figure 1A; Table S1).

A two‐dose ChAdOx1‐S vaccine course was received by the majority of HSCT/CAR‐T (105, 53.0%) and HCW (54, 56.3%) groups. Almost all participants received an mRNA vaccine for their third dose (38 HSCT‐recipients, 100.0%; 70 HCWs, 98.6%).

### Binding antibody responses

Although the majority of HSCT/CAR‐T recipients had detectable anti‐RBD IgG following vaccination (post‐V1: 45, 55.5%; post‐V2: 114, 85.7%), significantly fewer had a titre of ≥400 AU/mL (post‐V1: 4, 4.9%; post‐V2: 75, 56.4%). Only 19 of the 33 individuals with a titre of <400 AU/mL after two vaccine doses, had antibody titres of >400 AU/mL after the third dose (57.6%), and four remained seronegative (12.1%).

After adjustment for multiple covariates, post‐V2 levels of ≥400 AU/mL in HSCT/CAR‐T recipients were less likely with older age (compared with <45 year olds, odds ratio [OR] 0.05 and 95% confidence interval [95% CI] 0.00–0.44, *p* = 0.025 for 45–64 year olds; OR 0.03 and 95% CI 0.00–0.35, *p* = 0.015 for ≥65 years olds) and underlying lymphoid malignancy (OR 0.07, 95% CI 0.00–0.56, *p* = 0.036; Table S2). Only weak correlations were seen between V1–V2 dosing interval and post‐V2 anti‐RBD IgG level for mRNA vaccinees (*R* = 0.27, *p* = 0.06) and ChAdOx1‐S vaccinees (*R* = 0.24, *p* = 0.034; Figure S1).

### Neutralising antibody responses against ancestral SARS‐CoV‐2

**FIGURE 2:**
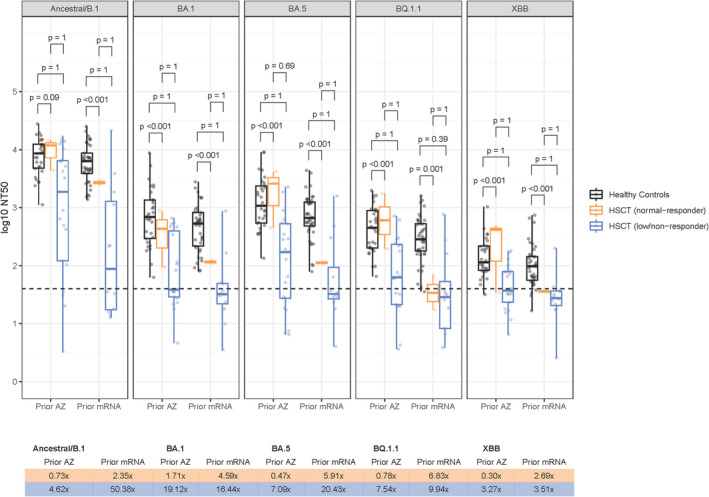
Data comparing HSCT‐recipients with healthy controls following a third vaccine dose (BNT162b2 or mRNA‐1273) split by whether they received ChAdOx1‐S (AZD1222; AZ) or mRNA for their first two doses. HSCT‐recipients are stratified by those with a low/no response (<400 AU/mL on anti‐RBD IgG, Roche) or normal response (>300 BAU mL − 1 Anti‐Spike IgG, Meso Scale Discovery) following two doses of either mRNA or ChAdOx1‐S vaccination. One healthy control received ChAdOx1‐S for their third dose therefore is not shown in this graph. *Y*‐axes denote log10 reciprocal dilution at which 50% neutralisation was observed (NT50). Boxplots demonstrate median and interquartile ranges (IQR25 and IQR75), with whiskers showing the range. Fold changes of the median for normal and low/non‐responder HSCT‐recipients compared with healthy controls are displayed in the table, whilst statistical comparisons are displayed above the box plots (Mann–Whitney *U* test and Bonferroni correction for multiple comparisons). Dashed line shows an NT50 of 40, considered the threshold at which true neutralising activity in serum is detected. HSCT, haematopoietic stem cell transplant. [Colour figure can be viewed at wileyonlinelibrary.com]

Prevaccination neutralising activity (NT50 ≥ 40) in sera against ancestral SARS‐CoV‐2 was seen in SARS‐CoV‐2 convalescent HCWs only, with limited or absent activity against Omicron lineage pseudoviruses (Figure 1A). HSCT/CAR‐T recipients were less likely to have neutralising activity in sera against the vaccine antigen ancestral SARS‐CoV‐2 compared with HCWs following their first two vaccinations (25% vs. 94.1% post‐V1, *p* < 0.001; 71.0% vs. 100.0% post‐V2, *p* < 0.001; Table S3). Quantitative nAb titres were also lower in HSCT/CAR‐T recipients than HCWs, with a greater difference after one dose (167‐fold, *p* < 0.001) than two doses (7‐fold, *p* < 0.001; Table S4; Figure 1B). Following a third dose, normal‐responder HSCT/CAR‐T recipients had similar neutralising activity to HCWs (Figure 2; Tables S3 and S4). In contrast, post‐V3 sera from low/non‐responders were less likely to neutralise ancestral SARS‐CoV‐2 than HCWs (*p* < 0.001; Table S3) and median nAb titres were 11‐fold lower than in HCWs (Table S4; *p* < 0.001).

**FIGURE 3:**
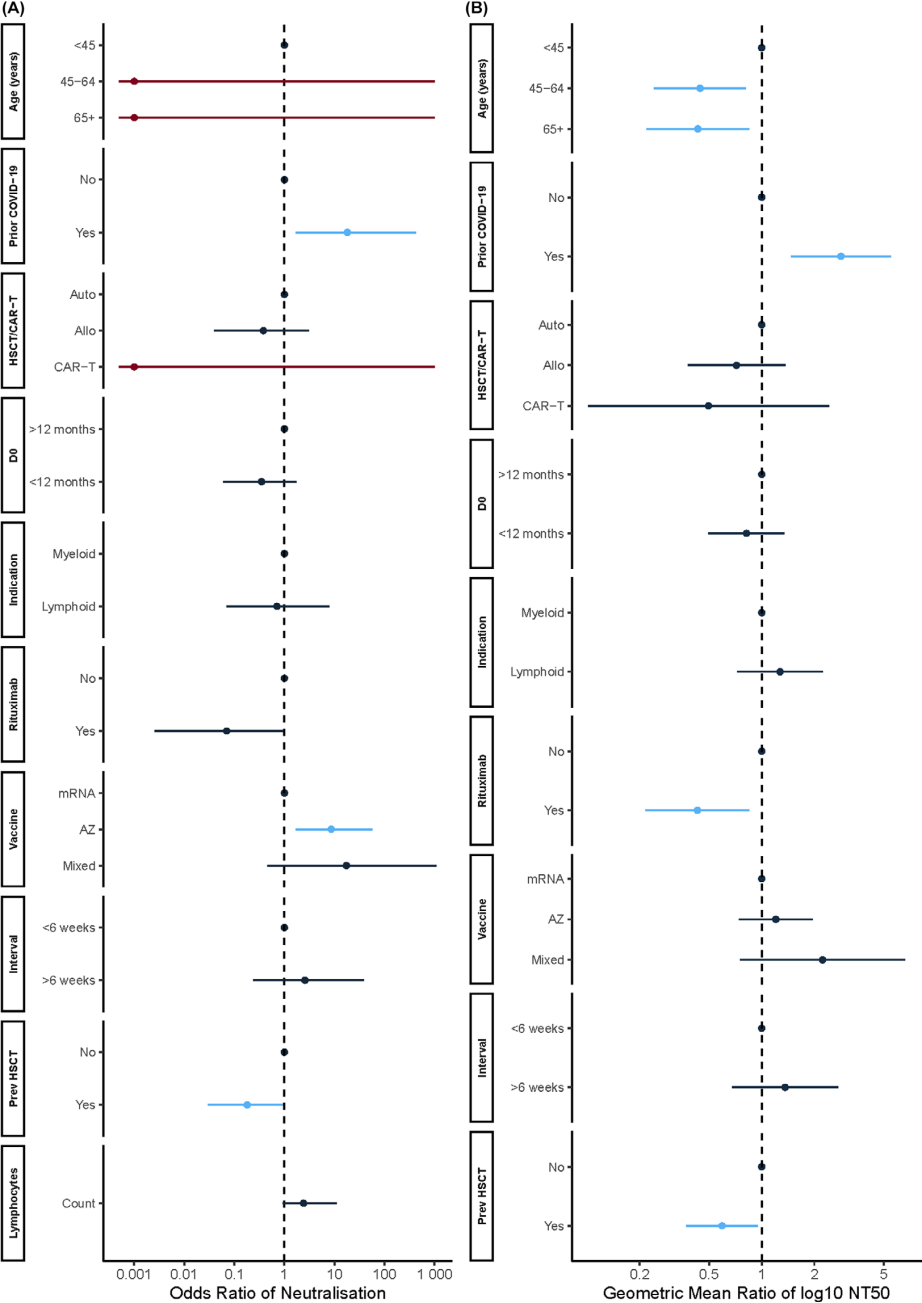
Forest plots from multivariable regression analyses demonstrating factors that impact neutralising antibody (nAb) responses after two SARS‐CoV‐2 vaccine doses in HSCT/CAR‐T recipients to the vaccination target ancestral SARS‐CoV‐2 (B.1). (A) Adjusted odds ratio and 95% confidence intervals (CI) from multivariable logistic regression exploring covariates that predict the presence of neutralising activity in serum. Variables with *p*‐values < 0.05 are shown in light blue, whilst burgundy represents factors with outlier odds ratios with incalculable confidence intervals as per Table S3 (B) Covariates impacting nAb titre following a second SARS‐CoV‐2 vaccine dose from a multivariable linear regression. Shown are geometric mean differences in log10 reciprocal dilution at which 50% neutralisation was observed (NT50) between the reference group (1.0) and other factors for each variable, along with 95% CI. Variables with *p*‐values < 0.05 are shown in light blue. In both cases, factors with a *p*‐value < 0.1 in univariate regression or those with an a priori hypothesis of impacting nAb were taken forward to multivariable analyses (Tables S6 and S7). Allo, allogeneic HSCT; Auto, autologous HSCT; AZ, ChAdOx1‐S vaccine (AZD1222); CAR‐T, chimeric antigen receptor T cell; HSCT D0, time between HSCT/CAR‐T and first vaccine dose; HSCT, haematopoietic stem cell transplant; Indication, type of haematological malignancy; Interval, interval between first and second vaccine doses; Lymph, total lymphocyte count (×10^9^/L) at the time of second vaccination (±7 days); Prev HSCT, previous HSCT prior to most recent HSCT/CAR‐T; Prior COVID‐19, history or serological evidence of prior COVID‐19 prior to vaccination; Rituximab, receipt of Rituximab in 12 months prior to recruitment. [Colour figure can be viewed at wileyonlinelibrary.com]

After adjustment for other factors, receiving ChAdOx1‐S (OR 8.63, 95% CI 1.67–57.69, *p* = 0.015) and prior COVID‐19 infection (OR 18.15, 95% CI 1.68–424.46, *p* = 0.034) increased the likelihood of HSCT/CAR‐T recipients having neutralising activity after two doses (Figure 3A; Table S5). Prior COVID‐19 infection was also associated with higher nAb titres (+1.04 log10 NT50, *p* = 0.003; Table S6; Figure 3B). HSCT/CAR‐T recipients who had undergone a previous HSCT were both less likely to have neutralising activity (OR 0.18, 95% CI 0.03–0.93, *p* = 0.049) and have lower nAb titres (−0.52 log10 NT50, *p* = 0.031), whilst older age (−0.81 log10 NT50, *p* = 0.009 for 45–64 years; −0.84 log10 NT50, *p* = 0.016 for ≥65 years; compared with aged <45 years) and receipt of rituximab (−0.85 log10 NT50, *p* = 0.016) were both associated with lower nAb titres (Table S6; Figure 3B). In allogeneic HSCT recipients, lymphocyte depletion with anti‐thymocyte globulin (ATG) reduced the likelihood of having neutralising activity (OR 0.00, 95% CI 0.00–0.09, *p* = 0.044; Table S5).

**FIGURE 4:**
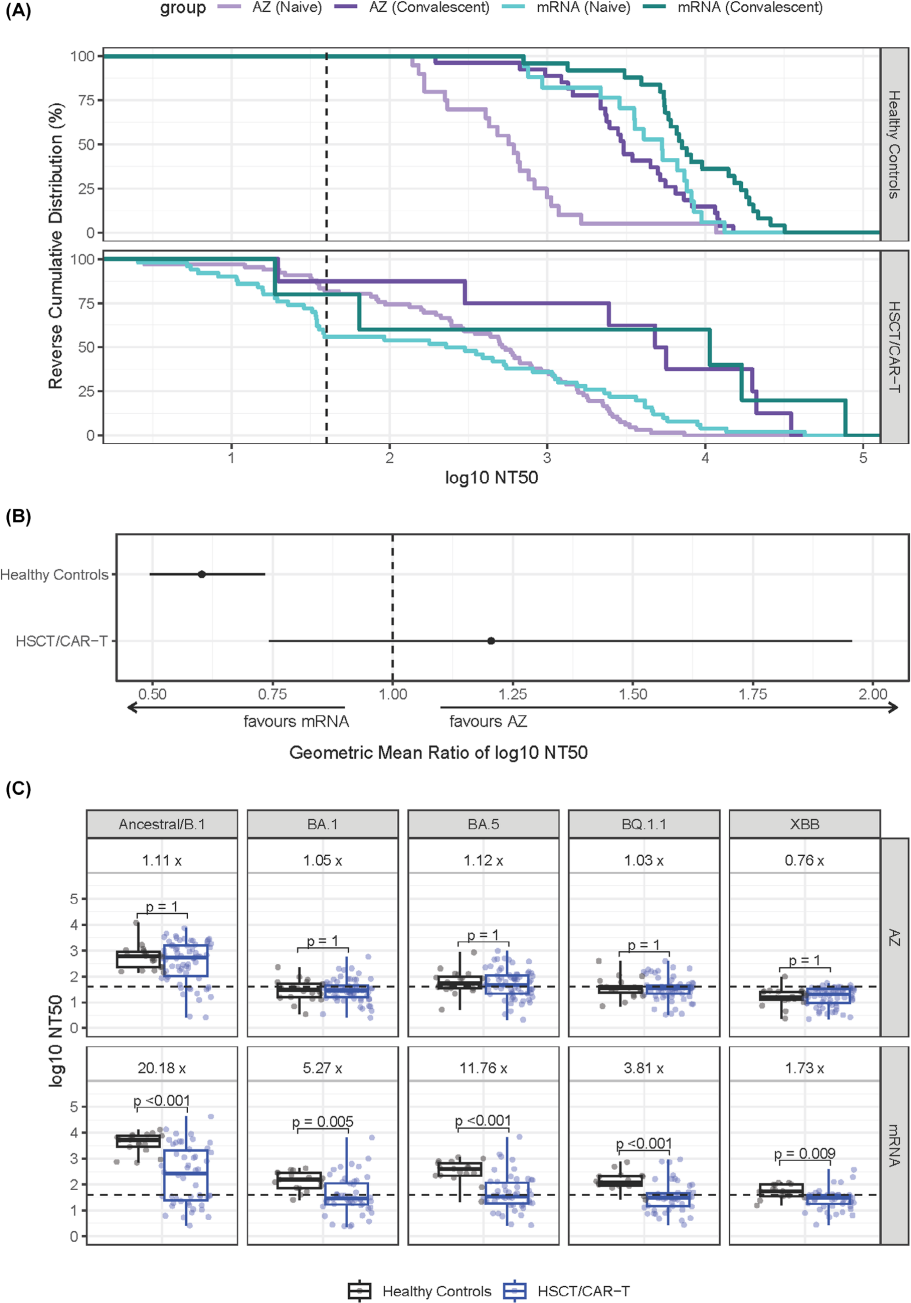
Comparison of neutralising antibody responses following a two‐dose SARS‐CoV‐2 vaccine course with ChAdOx1‐S (AZD1222; AZ) vaccine or mRNA vaccines (BNT162b2 or mRNA‐1273) in HSCT/CAR‐T recipients and healthy control participants. (A) Reverse cumulative plots of neutralising antibody (nAb) titres in healthy controls (top panel) and healthy controls (bottom panel), stratified by individuals who were naive or convalescent on SARS‐CoV‐2 anti‐nucleocapsid IgG testing prior to the first SARS‐CoV‐2 vaccine dose. NAb titres are expressed as the log10 reciprocal dilution at which 50% neutralisation was observed (NT50). Shown are the percentage of participants (*y* axis) with nAb activity above NT50 thresholds (*x*‐axis) against SARS‐CoV‐2 ancestral/B.1 pseudotype virus. Dashed line shows an NT50 of 40, considered the threshold at which true neutralising activity in serum is detected. Reverse cumulative plot created using stat_ecdf within ggplot2 package in R. (B) The geometric mean ratio (and 95% confidence intervals) of log 10 NT50 against ancestral/B.1 SARS‐CoV‐2 between individuals receiving ChAdOx1‐S and mRNA vaccine courses, adjusted for other covariates in multivariable linear regression analyses. (C) Comparison of nAb titres in SARS‐CoV‐2 naive healthy controls and HSCT/CAR‐T recipients, stratified by receipt of ChAdOx1‐S (top panel) or mRNA (bottom panel) vaccine courses. Shown are NT50 values (*Y*‐axes) against ancestral/B.1 and Omicron lineage viruses (BA.1, BA.5, BQ.1.1, XBB). Boxplots demonstrate median and interquartile ranges (IQR25 and IQR75), with whiskers showing the range. Fold change of the median absolute NT50 values and statistical comparisons between HSCT/CAR‐T recipients and healthy controls are displayed (Mann–Whitney *U* test with Bonferroni correction). Dashed line shows an NT50 of 40, considered the threshold at which true neutralising activity in serum is detected. The HSCT groups in the plots include both HSCT and CAR‐T recipients. CAR‐T, chimeric antigen receptor T cell; HSCT, haematopoietic stem cell transplant. [Colour figure can be viewed at wileyonlinelibrary.com]

In HCWs, after adjusting for other covariates, post‐V2 nAb titres were significantly lower in those vaccinated with ChAdOx1‐S compared with mRNA vaccines (−0.51 log10 NT50, *p* < 0.001, Table S6). This difference was not observed in HSCT/CAR‐T recipients (*p* = 0.455; Figure 3A,B; Table S6). In infection naïve participants, nAb titres following two doses of ChAdOx1‐S were similar in HSCT/CAR‐T recipients and HCWs (1.11‐fold, *p* = 1.00), but were significantly higher in HCW compared with HSCT/CAR‐T recipients in mRNA vaccinees (20‐fold, *p* < 0.001; Figure 4). Compared with HCWs, post‐V3 nAb titres in low/non‐responder HSCT‐recipients were 50‐fold lower for those who received mRNA for V1‐2 (*p* < 0.001), but only 4.6‐fold lower in those who received ChAdOx1‐S (*p* = 0.090; Figure 2).

### NAb responses against omicron SARS‐CoV‐2 lineages

HSCT/CAR‐T recipients were significantly less likely to neutralise the selected Omicron pseudotypes following their first two vaccinations than HCWs (Table S3), with NAb titres up to 10‐fold lower post‐V2 (BA.1 4.5‐fold, *p* < 0.001; BA.5 10.0‐fold, *p* < 0.001; BQ.1.1 4.4‐fold, *p* < 0.001; XBB 1.8‐fold, *p* < 0.001; Table S4). Larger fold change differences between HSCT/CAR‐T recipients and HCWs were also observed in mRNA vaccinees than ChAdOx1‐S vaccinees (Figure 3C; Figure S2). Low/non‐responder HSCT/CAR‐T recipients after two doses were less likely to have anti‐Omicron neutralising activity post‐V3 than HCWs (*p* < 0.001 for all lineages, Table S3). Although numbers were small, normal‐responder HSCT/CAR‐T recipients had more similar post‐V3 anti‐Omicron nAb titres to HCWs (Tables S3 and S4).

### NAb responses and COVID‐19

25 HSCT/CAR‐T recipients were reported to have had COVID‐19 following their second vaccination, with varying severity (asymptomatic *n* = 4, symptomatic but not hospitalised *n* = 5, hospitalised with no oxygen *n* = 3, intensive care *n* = 1, unknown *n* = 12). We did not detect a significant difference in nAbs between those who had a post‐V2 COVID‐19 infection and those who did not (Figures S4–S6).

### T‐cell responses post‐V2

No factors predicted the likelihood of HSCT/CAR‐T recipients developing a T‐cell response following two doses of vaccination, although numbers were limited (*n* = 44; Table S7). No differences in median SFC/10^6^ PBMCs were observed between vaccine groups (*p* = 0.58; Figure S3).

## DISCUSSION

Using participants recruited to several multicentre studies in the UK, we demonstrate that HSCT/CAR‐T recipients have lower SARS‐CoV‐2 nAb responses compared with healthy controls following a primary vaccination course. This difference is more marked after one dose compared with two, highlighting the need for at least two vaccine doses post‐HSCT/CAR‐T to generate any appreciable nAb response. A particularly interesting and novel finding was that HSCT/CAR‐T recipients receiving two ChAdOx1‐S vaccines were more likely to have anti‐SARS‐CoV‐2 nAb than those who received mRNA vaccines. In infection naïve participants, nAb titres between HSCT/CAR‐T recipients and HCWs receiving ChAdOx1‐S were remarkably similar, in contrast to the much higher titres seen in healthy adults following mRNA vaccines. Given that new HSCT/CAR‐T recipients have their immune memory ablated, this finding is particularly relevant when considering how to improve vaccine regimens in these groups.

Multiple studies demonstrate that mRNA vaccination results in enhanced humoral responses compared with other vaccine platforms in healthy individuals and other immunosuppressed groups.^5^, ^7^, ^13^, ^14^, ^15^, ^16^, ^17^, ^22^, ^23^ There is however a paucity of published data in HSCT/CAR‐T recipients for any vaccines other than homologous mRNA regimens.^24^, ^25^ In two recent meta‐analyses, only 111/5906 (1.9%) and 79/2899 (2.7%) HSCT/CAR‐T recipients received a homologous viral vector vaccine course, with a further 22/5906 (0.4%) and 12/2899 (0.4%) receiving a mixed ChAdOx1‐S/mRNA regimen.^17^, ^24^, ^25^, ^26^, ^27^, ^28^, ^29^, ^30^, ^31^, ^32^, ^33^, ^34^, ^35^, ^36^ One previously reported study of HSCT/CAR‐T recipients receiving ChAdOx1‐S (*n* = 34/55, 61.8%) found that allogeneic HSCT‐recipients receiving a single dose of ChAdOx1‐S were more likely to have detectable binding antibodies than those receiving a single dose of BNT162b2.^26^ Analysis of the entire cohort of clinically vulnerable adults in OCTAVE also demonstrated that ChAdOx1‐S was more likely to induce T‐cell responses.^7^ Our data represent the largest comparison of ChAdOx1‐S and mRNA primary vaccine courses in a single study of HSCT/CAR‐T recipients to date.

It is currently unclear why the SARS‐CoV‐2 nAb responses appear preserved in HSCT/CAR‐T recipients receiving ChAdOx1‐S compared with mRNA vaccines. In healthy individuals, differences in the immune response following ChAdOx1‐S and mRNA vaccinations have been observed.^37^ For instance, following a single dose, ChAdOx1‐S induces a memory‐like circulating T follicular helper cell and plasmablast response, whereas BNT162b2 does not.^37^ Further mechanistic studies are required to carefully dissect whether the different pathways of immune induction after viral vector and mRNA vaccines are affected differently by HSCT/CAR‐T‐therapy.^38^ In addition, as ChAdOx1‐S and other viral vector vaccines are no longer available in most countries, our findings should encourage the evaluation of other viral vector platform vaccines in development in these patient groups. We also observed that priming with ChAdOx1‐S vaccines in HSCT/CAR‐T low/non‐responders to two doses resulted in higher nAb titres after an mRNA 3rd dose, compared with those primed with an mRNA two‐dose course. A heterologous prime‐boost regimen is therefore worth evaluating in future clinical trials in HSCT/CAR‐T recipients.

UK guidelines for vaccinating severely immunosuppressed adults considered vaccine naive diverged in 2023 from other (inter‐) national guidelines, from three doses of mRNA vaccination (0, 3, and 7–16 weeks), to two doses (0 and 8–12 weeks) with a third dose during the next seasonal campaign.^5^, ^10^, ^39^, ^40^ Although the guidance allows the interval between the first two doses to be reduced to 3 weeks on specialist advice, a preference for a longer dosing interval is stated due to findings in healthy adults showing greater post‐dose two antibody.^5^, ^6^ We find that a longer dosing interval did not result in a statistically significant increase in neutralising activity in HSCT/CAR‐T recipients when adjusted for other factors. Previously published data in allogeneic HSCT‐recipients also suggest that a similar proportion have detectable binding antibodies with a short (21 days, 86%) and a long dosing interval (median 76 days, 86%).^7^, ^41^ Given that we and others have found that humoral responses are very poor in HSCT/CAR‐T recipients following a single SARS‐CoV‐2 vaccination, adhering to a minimum licensed interval between first and second doses would offer the best course of action for individual patients.^26^, ^28^, ^41^, ^42^, ^43^

Our study has several limitations, including small numbers in some groups (e.g. CAR‐T recipients, post‐V2 COVID‐19 severity data, T‐SPOT data) limiting the power to identify true differences. Our data were generated using SARS‐CoV‐2 ancestral vaccines, whilst Omicron lineage monovalent vaccines are currently in use. Nevertheless, our main findings are focused on vaccine‐matched nAb to ancestral SARS‐CoV‐2, therefore, we would expect HSCT/CAR‐T recipients receiving Omicron lineage vaccines to generate similar vaccine‐matched Omicron‐specific nAb data. Our data are observational and may not have accounted for unknown confounders affecting differences in groups.

Whilst COVID‐19 is now a mild infection for many individuals with considerable immune memory from prior infections and vaccines, new HSCT/CAR‐T recipients remain extremely immune‐vulnerable. Randomised clinical trials of new SARS‐CoV‐2 vaccines should consider dosing intervals and vaccine platforms to prioritise these and other immunosuppressed groups to reduce the gap in protective anti‐SARS‐CoV‐2 immunity.

## AUTHOR CONTRIBUTIONS

HC selected and tested samples on the pseudotype neutralisation assay, formally and visually analysed the data, and wrote the original draft. NT developed the pseudotype neutralisation assay and supplied the SARS‐CoV‐2 pseudotype plasmids. TIdS supervised the project and adapted the pseudoneutralisation assay with NB. RS, AP, BK, NB, HH, AA, IG, and SHL collated and shipped participant samples. BBL wrote the bespoke R script to analyse the luminescence files and generate NT50 values. AK, BK, and SHL curated the metadata. IBM, EB, CSG, PK, AR, SHL, TIdS, JAS, KO, PM, SJD, and PJC conceptualised one or more of the main studies. IBM (OCTAVE, OCTAVE‐DUO), PK (OCTAVE‐DUO), SHL (PROSECO), SJD (PITCH), and PJC (HERO) were chief investigators in the main studies. EB, TIdS, JAS, CSG, AR, DT, GC, IBM, MW, SS, KO, JC, EH, RM, MK, FK, RS, VP, KLY, DR, HP, and SB contributed to patient recruitment and data collection. All authors reviewed the manuscript (except PM who died in October 2022).

## CONFLICT OF INTEREST STATEMENT

SHL has received speaker's honoraria and is on an advisory board for AstraZeneca. JAS declares honoraria for educational events from Jazz, Gilead, Janssen, for advisory board membership from Medac, Vertex, Jazz, and BMS, and for trial IDMC membership from Kiadis Pharma.

## Supporting information


Appendix S1.

Appendix S2.

## Data Availability

Data are not publicly available due to datasets containing information that could compromise research participant privacy/consent. Reasonable requests for data may be considered as per the processes for each study as previously described.^7^, ^8^, ^9^, ^18^ R scripts used for regression and graphical analyses are publicly available, alongside a dummy dataset in order to run the code (https://github.com/xhayles/hsct‐cart‐covid‐pseudoneutralisation/).
