# Supplementary material for: Greater preservation of SARS‐CoV‐2 neutralising antibody responses following the ChAdOx1‐S (AZD1222) vaccine compared with mRNA vaccines in haematopoietic cell transplant recipients
Source: Br J Haematol. 2024 Nov 17;205(6):2206–18. doi: 10.1111/bjh.19874 (PMC11637739; doi:10.1111/bjh.19874)
Supplement: Supplementary file 1 — Appendix S1. [file BJH-205-2206-s001.docx]

| **Timepoint** | **HSCT/CAR-T recipients** | **Healthy controls** |
| --- | --- | --- |
| **Pre-vaccination samples**^1^   - n - Days post vaccination, median (range) | 0  NA | 59  NA |
| **Post-V1 samples**   - n - Days post vaccination, median (range) | 72  29 (10-80) | 51  28 (21 - 38) |
| **Post-V2 samples**   - n - Days post vaccination, median (range) | 138  29 (10-80) | 89  29 (21 - 41) |
| **Post-V3 samples**^2^   - n - Days post vaccination, median (range) | 38  21 (20-62) | 71  30 (21-96) |
| **Total** | **248** | **245** |

**Table S1. Sera tested on the pseudotype neutralisation assay by time point and time since vaccination.**

1. 33 were ‘convalescent’ and 26 were ‘naive’ on SARS-CoV-2 anti-nucleocapsid IgG assays Meso Scale Discovery (Angyal et. al. Lancet Microbe 2022) or in-house testing (Colton et al. Wellcome Open Res 2022). Sera were collected between 15 May 2020 to 18 February 2021.
2. For the HSCT-recipients, low/non-responders n=33 (OCTAVE-DUO) and normal responders n=5 (UK PROSECO).

*CAR-T: chimeric antigen receptor T cells, HSCT: Haematopoetic Stem Cell Transplant, V1: first vaccination, V2: second vaccination, V3: third vaccination.*

|  | **Univariate logistic regression**  (all HSCT/CAR-T recipients) | | | **Multivariable model 1**  (all HSCT/CAR-T recipients) | | | **Multivariable model 2**  (allogeneic-HSCT only) | | |
| --- | --- | --- | --- | --- | --- | --- | --- | --- | --- |
| Continuous factors | OR | 95% CI | P value | OR | 95% CI | P value | OR | 95% CI | P value |
| **Interval between V2 and post-V2 visit**, days | 0.99 | 0.96-1.02 | 0.589 | ND | ND | ND | ND | ND | ND |
| **Lymphocyte count at post-V2 visit** | **2.81** | **1.44-6.31** | **0.006** | 3.54 | 1.15-14.45 | 0.055 | 5.63 | 1.18-48.14 | 0.080 |
| **IgG level at post-V2 visit** | 0.98 | 0.83-1.15 | 0.773 | ND | ND | ND | ND | ND | ND |
| Discrete factors | OR | 95% CI | P value | OR | 95% CI | P value | OR | 95% CI | P value |
| **Age**, years   - <45 - 45-64 - ≥65 | -  **0.47**  **0.33** | -  **0.19-1.10**  **0.12-0.89** | -  **0.090**  **0.031** | -  **0.05**  **0.03** | -  **0.00-0.44**  **0.00-0.35** | -  **0.025**  **0.015** | **-**  **0.02**  **0.02** | **-**  **0.00-0.29**  **0.00-0.29** | **-**  **0.017**  **0.014** |
| **Sex**   - Female - Male | -  0.96 | -  0.48-1.93 | -  0.918 | -  ND | -  ND | -  ND | -  ND | -  ND | -  ND |
| **Initial course (V1 and V2)**   - mRNA^1^ - ChAdOx1-S - Mixed | -  1.25  0.85 | -  0.61-2.56  0.03-0.22 | -  0.539  0.911 | -  1.17  13.19 | -  0.31-4.53  0.27-1116.33 | -  0.815  0.191 | -  0.43  14.38 | -  0.05-2.68  0.22-2059.91 | -  0.393  0.225 |
| **Dosing interval**   - <6 weeks - >6 weeks | -  3.25 | -  0.86-15.63 | -  0.099 | -  1.48 | -  0.14-35.42 | -  0.761 | -  1.86 | -  0.06-104.47 | -  0.729 |
| **Evidence of COVID-19 prior to post-V2 visit^2^**   - Yes | 2.08 | 0.65-7.91 | 0.238 | NA | NA | 0.994 | NA | NA | 0.994 |
| **HSCT type**   - Autologous - Allogeneic - CAR-T | -  0.58  0.30 | -  0.25-1.32  0.05-1.45 | -  0.205  0.141 | -  0.65  0.24 | -  0.11-3.79  0.01-5.18 | -  0.631  0.380 | -  ND | -  ND | -  ND |
| **Interval between D0 and V1**   - >12 months - <12 months | **-**  **0.21** | **-**  **0.10-0.45** | **-**  **<0.001** | -  0.53 | -  0.13-1.99 | -  0.355 | -  0.19 | -  0.02-1.28 | -  0.120 |
| **Indication**^3^   - Myeloid - Neurological - Lymphoid - Other | -  3.85  0.62  NA | -  0.59-75.53  0.28-1.35  NA | -  0.227  0.227  0.987 | **-**  ND  **0.07**  ND | **-**  ND  **0.00-0.56**  ND | **-**  ND  **0.036**  **ND** | -  ND  0.06  ND | -  ND  0.00-0.73  ND | -  ND  0.052  ND |
| **Disease status (if applicable)**   - Remission - Partial remission - Progressive or relapsed disease - Stable disease | -  0.50  0.30  0.93 | -  0.12-1.86  0.04-1.46  0.23-3.98 | -  0.304  0.161  0.920 | -  ND | -  ND | -  ND | -  ND | -  ND | -  ND |
| **Conditioning**^4^   - Reduced intensity - Myeloablative - Non-myeloablative | -  0.85  0.95 | -  0.33-2.77  0.21-3.42 | -  0.919  0.817 | -  ND | -  ND | -  ND | -  0.12  0.26 | -  0.00-1.78  0.00-5.59 | -  0.150  0.453 |
| **Lymphocyte depletion**^4^   - Other^5^ - ATG - None | -  0.74  0.61 | -  0.27-2.02  0.11-3.04 | -  0.556  0.540 | -  ND | -  ND | -  ND | -  2.64  0.75 | -  0.26-37.98  0.02-14.79 | -  0.428  0.852 |
| **Total Body Irradiation**   - Yes | 1.96 | 0.70-6.03 | 0.215 | ND | ND | ND | ND | ND | ND |
| **Previous HSCT**   - Yes | 0.66 | 0.29-1.51 | 0.321 | ND | ND | ND | ND | ND | ND |
| **Rituximab receipt**   - Yes | **0.09** | **0.01-0.36** | **0.003** | NA | NA | 0.992 | NA | NA | 0.992 |
| **Any GVHD at V1 (if applicable)**   - Yes | 1.02 | 0.34-3.14 | 0.978 | ND | ND | ND | ND | ND | ND |
| **Any GVHD at V2 (if applicable)**   - Yes | 0.87 | 0.29-2.56 | 0.793 | ND | ND | ND | ND | ND | ND |

**Table S2. Logistic regression demonstrating factors affecting the likelihood of a normal binding antibody response in HSCT/CAR-T recipients following two doses of SARS-CoV-2 vaccines.** A normal binding antibody response is defined as ≥400 AU/ml on the Roche Elecsys anti-RBD IgG assay. Shown are univariate and multivariable logistic regression models including all HSCT/CAR-T recipients (including all participants where data available for each variable), in addition to a multivariable logistic regression model for allogeneic-HSCT recipients only to include data collected for his group only such as conditioning regimen. Factors that are statistically significant at p<0.05 are highlighted in red, with factors with a p value of 0.05-0.09 are highlighted in orange. Factors that either had a p value <0.1 on univariate analysis or an *a priori* hypothesis were taken forward to multivariable logistic regression.

1. mRNA includes BNT162b2 (BioNTech Pfizer) and mRNA-1273 (Moderna)
2. Determined by either positive Roche Elecsys AntiSARS-CoV-2 nucleocapsid assay or reported PCR confirmed COVID-19.
3. As the ‘neurological’ and ‘other’ groups all received ChAdOx1-S vaccination, for the multivariable analysis they were marked “NA” in the metadata to allow for comparison of ‘lymphoid’ to ‘myeloid’ whilst correcting zero cells in the contingency table.
4. Although conditioning and lymphocyte depletion are *a priori* factors, these variables were only assessed in multivariable model 2 as data were only available for allogeneic HSCT-recipients.
5. Other includes campath or post-transplant cyclophosphamide.

*ATG: Anti-thymocyte globulin, CAR-T: chimeric antigen receptor T cells, D0: Day zero, GVHD: Graft versus host disease, HSCT: Haematopoetic Stem Cell Transplant, NA: Not applicable, ND: Not done, RBD: Receptor binding domain, V1: first vaccination, V2: second vaccination.*

|  | **Ancestral/B.1** | **BA.1** | **BA.5** | **BQ.1.1** | **XBB** |
| --- | --- | --- | --- | --- | --- |
| **Post-V1**  HC, n (%)  HSCT, n (%)  *P value*  *OR*  *95% CI* | 48/51 (94.1)  18/72 (25.0)  **<0.001**  **0.022**  **0.004-0.079** | 32/51 (62.7)  5/72 (6.9)  **<0.001**  **0.046**  **0.012-0.139** | 37/51 (72.5)  6/72 (8.3)  **<0.001**  **0.036**  **0.010-0.106** | 36/51 (70.6)  3/72(4.2)  **<0.001**  **0.019**  **0.003-0.071** | 31/51 (60.8)  5/72 (6.9)  **<0.001**  **0.050**  **0.013-0.151** |
| **Post-V2**  HC, n (%)  HSCT, n (%)  *P value*  *OR*  *95% CI* | 89/89 (100.0)  98/138 (71.0)  **<0.001**  **0.000**  **0.000-0.111** | 69/89 (77.5)  48/138 (34.8)  **<0.001**  **0.156**  **0.080-0.295** | 80/89 (89.9)  70/138 (50.7)  **<0.001**  **0.117**  **0.048-0.257** | 71/89 (79.8)  47/138 (34.1)  **<0.001**  **0.132**  **0.066-0.254** | 53/89 (59.6)  21/138 (15.2)  **<0.001**  **0.123**  **0.062-0.239** |
| **Post-V3 (NR)**  HC, n (%)  HSCT, n (%)  *P value*  *OR*  *95% CI* | 71/71 (100.0)  5/5 (100.0)  1.000  1.000  NA | 71/71 (100.0)  5/5 (100.0)  1.000  1.000  NA | 71/71 (100.0)  5/5 (100.0)  1.000  1.000  NA | 70/71 (98.6)  4/5 (80.0)  0.128  0.063  0.001-5.599 | 66/71 (93.0)  2/5 (40.0)  **0.007**  **0.055**  **0.004-0.593** |
| **Post-V3 (LR)**  HC, n (%)  HSCT, n (%)  *P value*  *OR*  *95% CI* | 71/71 (100.0)  23/33 (69.7)  **<0.001**  **0.000**  **0.000-0.162** | 71/71 (100.0)  14/33 (42.4)  **<0.001**  **0.000**  **0.000-0.051** | 71/71 (100.0)  18/33 (54.5)  **<0.001**  **0.000**  **0.000-0.082** | 70/71 (98.6)  16/33 (48.5)  **<0.001**  **0.014**  **0.000-0.102** | 66/71 (93.0)  12/33 (36.4)  **<0.001**  **0.045**  **0.011-0.153** |

**Table S3. HSCT/CAR-T recipient and healthy control sera with an NT50 ≥40 (considered the threshold at which true neutralising activity in serum is detected) against each SARS-CoV-2 pseudotyped virus.** P values, odds ratio (OR) and 95% confidence intervals (95% CI) calculated using Fisher’s exact test. HSCT groups in the table include both HSCT and CAR-T recipients. Comparisons with a p value of <0.05 are highlighted in bold.

*CAR-T: Chimeric Antigen Receptor T-cell, HC: Healthy control, HSCT: Haematopoetic Stem Cell Transplant, LR: Low- or non-responder to two doses of vaccine, NR: Normal responder to two doses of vaccine, NT50: half-maximal neutralising titre, OR: Odds ratio.*

|  | **Ancestral/B.1** | **BA.1** | **BA.5** | **BQ.1.1** | **XBB** |
| --- | --- | --- | --- | --- | --- |
| **Post V1,** median NT50 (range)  Healthy controls  HSCT/CAR-T recipients  *Fold change of the median NT50*  *P value* | 4790 (2-26187)  29 (4-23855)  167.0×  **<0.001** | 118 (3-2049)  19 (2-3231)  6.3×  **<0.001** | 570 (7-4538)  23 (2-4586)  24.4×  **<0.001** | 350 (2-2935)  19 (2-1578)  18.4×  **<0.001** | 60 (8-843)  23 (2-287)  2.6×  **<0.001** |
| **Post V2,** median NT50 (range)  Healthy controls  HSCT/CAR-T recipients  *Fold change of the median NT50*  *P value* | 3568 (139-31884)  500 (3-77273)  7.1×  **<0.001** | 140 (3-2176)  32 (2-6494)  4.5×  **<0.001** | 410 (5-5086)  41 (2-18860)  10.0×  **<0.001** | 153 (7-3514)  35 (3-2651)  4.4×  **<0.001** | 50 (2-906)  28 (2-1316)  1.8×  **<0.001** |
| **Post V3 (NR),** median NT50 (range)  Healthy controls  HSCT-recipients (normal-responders)  *Fold change of the median NT50*  *P value* | 6936 (1126-27951)  4417 (2493-14588)  1.6×  1.000 | 575 (63-8802)  124 (93-890)  4.6×  1.000 | 775 (79 - 9070)  449 (106-4162)  1.7×  1.000 | 319 (36-1977)  194 (17-1763)  1.6×  1.000 | 100 (17-1034)  38 (34-470)  2.6×  1.000 |
| **Post V3 (LR),** median NT50 (range)  Healthy controls  HSCT-recipients (low/non-responders)  *Fold change of the median NT50*  *P value* | 6936 (1126-27951)  644 (3-21432)  10.8×  **<0.001** | 575 (63-8802)  37 (4-876)  15.4×  **<0.001** | 775 (79 - 9070)  64 (4-2298)  12.1×  **<0.001** | 319 (36-1977)  37 (4-761)  8.6×  **<0.001** | 100 (17-1034)  33 (3-200)  3.0×  **<0.001** |

**Table S4. Median half-maximal neutralising titres (NT50) in HSCT/CAR-T recipients and healthy controls for pseudotyped SARS-CoV-2 viruses.** Fold change of the median is calculated by dividing HC / HSCT absolute median NT50 values. Mann Whitney U test was used to calculate p values with Bonferroni adjustment. HSCT groups in the table include both HSCT and CAR-T recipients.

*CAR-T: chimeric antigen receptor T cells, HSCT: Haematopoetic Stem Cell Transplant, NR: Normal responder to two doses of vaccine, LR: Low/non responder to two doses of vaccine.*

|  | **Univariate logistic regression**  (all HSCT/CAR-T recipients) | | | **Multivariable model 1**  (all HSCT/CAR-T recipients) | | | **Multivariable model 2**  (allogeneic-HSCT only) | | |
| --- | --- | --- | --- | --- | --- | --- | --- | --- | --- |
| Continuous factors | OR | 95% CI | p value | OR | 95% CI | p value | OR | 95% CI | p value |
| **Interval between V2 and post-V2 visit**, days | 1.02 | 0.99- 1.07 | 0.299 | ND | ND | ND | ND | ND | ND |
| **Lymphocyte count at post-V2 visit** | **2.38** | **1.19-5.43** | **0.025** | 2.41 | 0.92-11.12 | 0.188 | 9.78 | 0.82-596.54 | 0.161 |
| **IgG level at post-V2 visit** | 0.97 | 0.82- 1.15 | 0.716 | ND | ND | ND | ND | ND | ND |
| Discrete factors | OR | 95% CI | p value | OR | 95% CI | p value |  |  |  |
| **Age, years**   - <45 - 45-64 - ≥65 | -  **0.21**  **0.21** | -  **0.05-0.69**  **0.05-0.75** | -  **0.019**  **0.003** | -  1.25e-09  7.00e-10 | -  NA  NA | -  0.993  0.993 | -  NA  NA | -  NA  NA | -  0.994  0.994 |
| **Sex**   - Female - Male | -  1.67 | -  0.80-3.53 | -  0.174 | -  ND | -  ND | -  ND | -  ND | -  ND | -  ND |
| **Initial course (V1 and V2)^1^**   - mRNA - ChAdOx1-S - Mixed | -  **3.22**  1.37 | -  **1.48-7.28**  0.12-30.49 | **-**  **0.004**  0.801 | -  **8.63**  17.29 | -  **1.67-57.69**  0.46-1095.86 | -  **0.015**  0.140 | -  **569.66**  2035.66 | -  **8.06-5.28e+06**  0.48-360.93 | -  **0.035**  0.074 |
| **Dosing interval between V1-2**   - <6 weeks - >6 weeks | -  2.71 | -  0.80-9.21 | -  0.103 | -  2.57 | -  0.24-38.96 | -  0.450 | -  3.36 | -  0.05-1333.31 | -  0.595 |
| **Evidence of COVID-19 prior to post-V2 visit^2^**   - Yes | 2.06 | 0.60-9.51 | 0.287 | **18.15** | **1.68-424.46** | **0.034** | 358.68 | 3.75-9.42e+06 | 0.078 |
| **HSCT type**   - Autologous - Allogeneic - CAR-T | -  0.36  **0.06** | -  0.11-0.95  **0.00-0.55** | -  0.054  **0.023** | -  0.38  1.58e-09 | -  0.04-3.05  NA | -  0.373  0.993 | -  ND | -  ND | -  ND |
| **Interval between D0 and V1**   - >12 months - <12 months | -  **0.16** | -  **0.06-0.40** | -  **<0.001** | -  0.35 | -  0.06-1.74 | -  0.200 | -  0.06 | -  0.00-1.36 | -  0.137 |
| **Indication**^3^   - Myeloid - Neurological - Lymphoid - Other | -  NA  0.91  NA | -  NA  0.40-2.15  NA | -  0.993  0.816  0.993 | -  ND  0.71  ND | -  ND  0.07-7.92  ND | -  ND  0.771  ND | -  ND  0.01  ND | -  ND  0.00-1.07  ND | -  ND  0.129  ND |
| **Disease status (if applicable)**   - Remission - Partial remission - Progressive or relapsed disease - Stable disease | -  0.98  0.56  0.84 | -  0.25- 4.77  0.12- 2.98  0.21- 4.18 | -  0.972  0.463  0.809 | -  ND | -  ND | -  ND | -  ND | -  ND | -  ND |
| **Conditioning**^4^   - Reduced intensity - Myeloablative - Non-myeloablative | -  1.14  0.75 | -  0.41-3.40  0.19-3.23 | -  0.802  0.682 | -  ND | -  ND | -  ND | -  4.20  0.00 | -  0.15-173.38  0.00-0.29 | -  0.401  0.078 |
| **Lymphocyte depletion**^4^   - Other^5^ - ATG - None | -  0.59  2.77 | -  0.22-1.56  0.43-54.34 | -  0.281  0.362 | -  ND | -  ND | -  ND | -  **0.00**  3.33 | -  **0.00-0.09**  0.25-93.54 | -  **0.044**  0.394 |
| **Total Body Irradiation**   - Yes | 1.20 | 0.45- 3.60 | 0.731 | ND | ND | ND | ND | ND | ND |
| **Previous HSCT**   - Yes | **0.36** | **0.16- 0.85** | **0.018** | **0.18** | **0.03-0.93** | **0.049** | **0.00** | **0.00-0.12** | **0.046** |
| **Rituximab receipt**   - Yes | **0.22** | **0.07- 0.67** | **0.008** | 0.07 | 0.00-0.96 | 0.073 | 0.00 | 0.00-0.36 | 0.079 |
| **Any GVHD at V1 (if applicable)**   - Yes | 1.63 | 0.52-6.22 | 0.429 | ND | ND | ND | ND | ND | ND |
| **Any GVHD at V2 (if applicable)**   - Yes | 2.02 | 0.58- 9.42 | 0.305 | ND | ND | ND | ND | ND | ND |

**Table S5. Logistic regression models demonstrating factors affecting the likelihood of developing neutralising antibodies against Ancestral/B.1 SARS-CoV-2 following two doses of vaccination in HSCT/CAR-T recipients.** The threshold for detection of neutralising activity is an NT50 ≥40 (considered the threshold at which true neutralising activity in serum is detected). Shown are univariate and multivariable logistic regression models including all HSCT/CAR-T recipients (including all participants where data available for each variable), in addition to a multivariable logistic regression model for allogeneic-HSCT recipients only to include data collected for his group only such as conditioning regimen. Factors that are statistically significant at p<0.05 are highlighted in red, with factors with a p value of 0.05-0.09 are highlighted in orange. Univariate and multivariable regression were used to calculate odds ratios (OR), 95% confidence interval (CI) and p values. Factors that either had a p value <0.1 on univariate analysis or an *a priori* hypothesis were taken forward to multivariable logistic regression.

1. mRNA includes BNT162b2 (BioNTech Pfizer) and mRNA-1273 (Moderna)
2. Determined by either positive Roche Elecsys AntiSARS-CoV-2 nucleocapsid assay or previous PCR confirmed COVID-19.
3. As the ‘neurological’ and ‘other’ groups all received ChAdOx1-S vaccination, for the multivariable analysis they were marked “NA” in the metadata to allow for comparison of ‘lymphoid’ to ‘myeloid’ whilst correcting zero cells in the contingency table.
4. Although conditioning and lymphocyte depletion are a priori factors, as data were only available for allogeneic HSCT-recipients, these variables were only assessed in multivariable model 2.
5. Other includes campath or post-transplant cyclophosphamide.
6. *ATG: Anti-thymocyte globulin, CAR-T: chimeric antigen receptor T cells, HSCT: Haematopoetic Stem Cell Transplant, NA: Not applicable, ND: Not done, OR: Odds ratio, V1: first vaccination, V2: second vaccination.*

|  | **HSCT/CAR-T recipients** | | | | | | **Healthy Controls** | | | |
| --- | --- | --- | --- | --- | --- | --- | --- | --- | --- | --- |
|  | **Univariate linear regression (all HSCT/CAR-T recipients)** | | **Multivariable model 1**  **(all HSCT/CAR-T recipients)** | | **Multivariable model 2 (allogeneic HSCT only)** | | **Univariate linear regression** | | **Multivariable regression** | |
| Continuous factors | Coefficient estimate | p value | Coefficient estimate | p value | Coefficient estimate | p value | Coefficient estimate | p value | Coefficient estimate | p value |
| **Interval between V2 and post-V2 visit**, days | 0.00 | 0.624 | ND | ND | ND | ND | ND | ND | ND | ND |
| **Lymphocyte count at post-V2 visit** | 0.22 | 0.082 | 0.13 | 0.272 | 0.12 | 0.367 | ND | ND | ND | ND |
| **IgG level at post-V2 visit** | 0.03 | 0.435 | ND | ND | ND | ND | ND | ND | ND | ND |
| Discrete factors | Coefficient estimate | p value | Coefficient estimate | p value |  |  | Coefficient estimate | p value | Coefficient estimate | p value |
| **Age**, years   - <45 - 45-64 - ≥65 | -  -0.39  **-0.56** | -  0.089  **0.023** | -  **-0.81**  **-0.84** | -  **0.009**  **0.016** | -  **-0.89**  **-0.91** | -  **0.021**  **0.036** | -  **0.43**  0.19 | -  **<0.001**  0.735 | -  0.16  0.07 | -  0.119  0.876 |
| **Sex**   - Female - Male | -  0.04 | -  0.836 | -  ND | -  ND | -  ND | -  ND | -  -**0.56** | -  **<0.001** | -  -0.14 | -  0.275 |
| **Initial course (V1 and V2)^1^**   - mRNA - ChAdOx1-S - Mixed | -  0.21  0.46 | -  0.241  0.448 | -  0.19  0.80 | -  0.445  0.147 | -  0.15  0.69 | -  0.612  0.245 | -  **-0.60**  NA | -  **<0.001**  NA | -  **-0.51**  NA | -  **<0.001**  NA |
| **Dosing interval between V1-2**   - <6 weeks - >6 weeks | -  0.54 | -  0.085 | -  0.31 | -  0.382 | -  0.20 | -  0.639 | -  -0.20 | -  0.427 | -  ND | -  ND |
| **Evidence of COVID-19 prior to post-V2 visit^2^**   - Yes | **0.81** | **0.004** | **1.04** | **0.003** | 0.80 | 0.074 | **0.559** | **<0.001** | **0.49** | **<0.001** |
| **HSCT type**   - Autologous - Allogeneic - CAR-T | -  -0.35  -0.81 | -  0.085  0.135 | -  -0.33  -0.70 | -  0.305  0.381 | -  ND | -  ND | - | -  NA | -  NA | -  NA |
| **Interval between D0 and V1**   - >12 months - <12 months | -  **-0.50** | -  **0.005** | -  -0.20 | -  0.427 | -  -0.24 | -  0.445 | -  NA | -  NA | -  NA | -  NA |
| **Indication**^3^   - Myeloid - Neurological - Lymphoid - Other | -  0.23  -0.02  1.03 | -  0.660  0.907  0.052 | -  ND  0.24  ND | -  ND  0.395  ND | -  ND  0.08  ND | -  ND  0.801  ND | -  NA | -  NA | -  NA | -  NA |
| **Disease status (if applicable)**   - Remission - Partial remission - Progressive or relapsed disease - Stable disease | -  0.28  -0.32  -0.13 | -  0.419  0.441  0.717 | -  ND | -  ND | -  ND | -  ND | -  NA | -  NA | -  NA | -  NA |
| **Conditioning**^4^   - Reduced intensity - Myeloablative - Non-myeloablative | -  -0.14  -0.17 | -  0.577  0.626 | -  ND | -  ND | -  -0.40  -0.08 | -  0.242  0.850 | -  NA | -  NA | -  NA | -  NA |
| **Lymphocyte depletion**^4^   - Other^5^ - ATG - None | -  -0.35  0.11 | -  0.160  0.798 | -  ND | -  ND | -  -0.36  -0.02 | -  0.304  0.973 | NA | NA | NA | NA |
| **Total Body Irradiation**   - Yes | -0.15 | 0.544 | ND | ND | ND | ND | NA | NA | NA | NA |
| **Previous HSCT**   - Yes | **-0.42** | **0.048** | **-0.52** | **0.031** | **-0.77** | **0.009** | NA | NA | NA | NA |
| **Rituximab receipt**   - Yes | **-0.85** | **0.003** | **-0.85** | **0.016** | -0.71 | 0.061 | NA | NA | NA | NA |
| **Any GVHD at V1 (if applicable)**   - Yes | 0.09 | 0.753 | ND | ND | ND | ND | NA | NA | NA | NA |
| **Any GVHD at V2 (if applicable)**   - Yes | -0.03 | 0.911 | ND | ND | ND | ND | NA | NA | NA | NA |

**Table S6. Linear regression demonstrating factors affecting the neutralising antibody titre (log10 NT50) against Ancestral/B.1 SARS-CoV-2 following two doses of vaccination in HSCT/CAR-T recipients and healthy controls.** Shown are univariate and multivariable linear regression models including all HSCT/CAR-T recipients (including all participants where data available for each variable), in addition to a multivariable linear regression model for allogeneic-HSCT recipients only to include data collected for his group only such as conditioning regimen. Factors that are statistically significant at p<0.05 are highlighted in red, with factors with a p value of 0.05-0.09 are highlighted in orange. Factors that either had a p value <0.1 on univariate analysis or an *a priori* hypothesis were taken forward for multivariable regression. Dosing interval in healthy controls was not taken forward to multivariable regression as numbers in the <6 week cohort were too small (n=3/89) and data were missing in 38/89. Coefficient estimates refer to log10 NT50 units.

1. mRNA includes BNT162b2 (BioNTech Pfizer) and mRNA-1273 (Moderna)
2. Determined by either positive Roche Elecsys AntiSARS-CoV-2 nucleocapsid assay or previous PCR confirmed COVID-19.
3. As the ‘neurological’ and ‘other’ groups all received ChAdOx1-S vaccination, for the multivariable analysis they were marked “NA” in the metadata to allow for comparison of ‘lymphoid’ to ‘myeloid’ whilst correcting zero cells in the contingency table.
4. Although conditioning and lymphocyte depletion are *a priori* factors, as data were only available for allogeneic HSCT-recipients, these variables were only assessed in multivariable model 2.
5. Other includes campath or post-transplant cyclophosphamide.

*ATG: Anti-thymocyte globulin, CAR-T: chimeric antigen receptor T cells, HSCT: Haematopoetic Stem Cell Transplant, NT50: Half maximal neutralising titres, OR: Odds ratio, V1: first vaccination, V2: second vaccination.*

|  | **Odds of having a detectable T-cell response^1^** | | |
| --- | --- | --- | --- |
| Factor | OR | 95% CI | p value |
| **Time since V2,** days | 0.95 | 0.80-1.10 | 0.493 |
| **Dosing interval between V1-V2,** days^2^ | 1.02 | 0.99-1.08 | 0.435 |
| **Lymphocyte count at post V2 visit** | 0.31 | 0.06-1.01 | 0.132 |
| Factor | OR | 95% CI | p value |
| **Age**  - <45  - 45-64  - ≥65 | -  1.00  2.33 | -  0.22-4.49  0.38-16.28 | -  1.000  0.365 |
| **Sex**   - Female - Male | -  1.07 | -  0.31-3.65 | -  0.911 |
| **Initial course (V1 and V2)^3^**   - mRNA - ChAdOx1-S | -  1.08 | -  0.30-3.79 | -  0.908 |
| **COVID-19 prior to post-V2 visit**   - No - Yes | -  0.81 | -  0.13-4.87 | -  0.810 |
| **HSCT-type**   - Autologous - Allogeneic - CAR-T | -  0.47  1.00 | -  0.10-1.83  0.07-25.45 | -  0.287  1.000 |
| **Interval between D0 and V1**   - >12 months - <12 months | -  0.65 | -  0.19-2.23 | -  0.495 |
| **Indication**   - Myeloid - Lymphoid - Neurological - Other | -  1.08  3.95e+07  2.18e-08 | -  0.29-4.19  NA  NA | -  0.906  0.994  0.996 |

**Table S7. Logistic regression to explore factors predicting post-V2 T-cell responses.**

1. Defined as >4 SFC/10^6^ PBMC.
2. Calculated as continuous as all >6 weeks.
3. mRNA includes BNT162b2 (BioNTech Pfizer) and mRNA-1273 (Moderna)

*CAR-T: chimeric antigen receptor T cells, CI: confidence interval, D0: Day zero, HSCT: Haematopoetic Stem Cell Transplant, OR: odds ratio, PBMC: peripheral blood mononuclear cells, SFC: spot-forming cells, V1: first vaccination, V2: second vaccination*

*
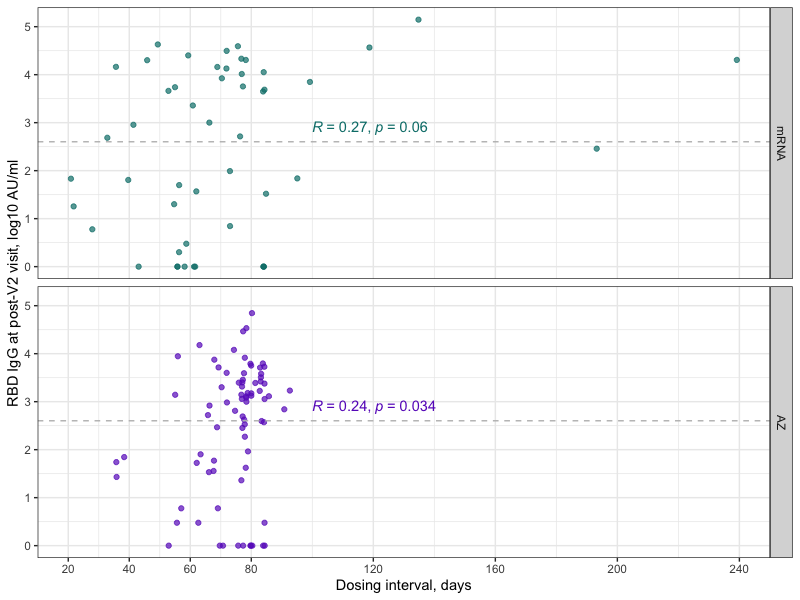
*

**Figure S1. Anti-SARS-CoV-2 anti-RBD IgG values in log10 AU/ml after the second vaccine dose for HSCT/CAR-T recipients plotted against the dosing interval between the first and second vaccine doses in days. Spearman’s correlation coefficient shown for mRNA (upper panel) and AZ ChAdOx1-S (lower panel) vaccinated groups.** Grey dashed line shows an anti-RBD IgG of 400AU/ml, considered the threshold of a normal response. mRNA includes BNT162b2 (BioNTech Pfizer) and mRNA-1273 (Moderna), and AZ refers to AstraZeneca ChAdOx1-S AZD1222.

*CAR-T: chimeric antigen receptor T cells, HSCT: Haematopoetic Stem Cell Transplant, RBD: Receptor binding domain, V1: first vaccination, V2: second vaccination. R = Spearman’s rho.*


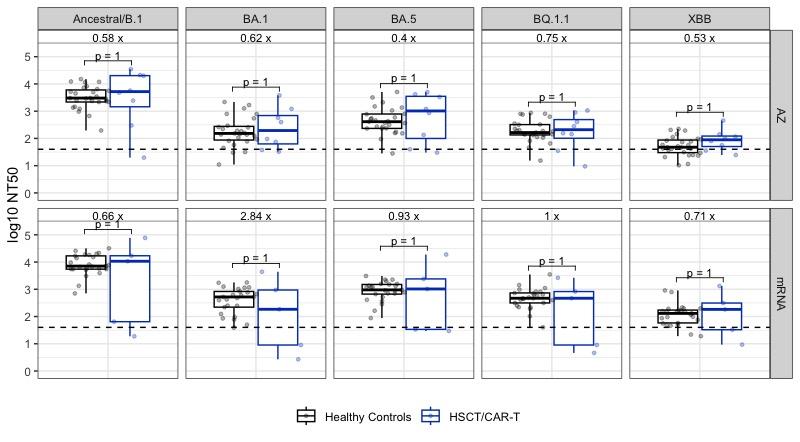


**Figure S2. Post-V2 NT50 values for convalescent HSCT/CAR-T recipients and healthy controls.** Split by pseudotype and vaccination type received. Fold change of the median absolute NT50 values are shown at the top of each facet. Boxplots demonstrate the median and interquartile ranges (IQR25 and IQR75), and the whiskers the range. P values calculated using the Mann Whitney U test with Bonferroni adjustment are shown above the box plots. mRNA includes BNT162b2 (BioNTech Pfizer) and mRNA-1273 (Moderna), and AZ refers to AstraZeneca ChAdOx1-S AZD1222. The HSCT groups in the plot include both HSCT and CAR-T recipients.

*CAR-T: Chimeric Antigen Receptor T-cell, HC: Healthy control, HSCT: Haematopoetic stem cell transplant, NT50: Half-maximal neutralising titre, V2: second vaccination*


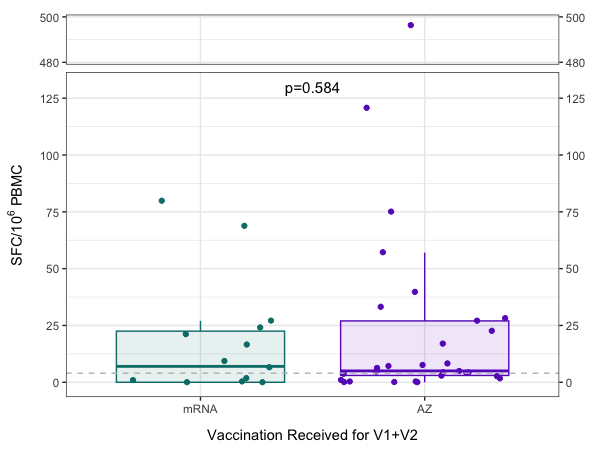


**Figure S3. Post-V2 T-SPOT responses in HSCT/CAR-T recipients (SFC/10^6^ PBMCs) split by vaccination type received for the first and second vaccine doses.** Boxplots demonstrate the median and interquartile ranges (IQR25 and IQR75), and the whiskers the range. P values calculated using the Mann Whitney U test with Bonferroni adjustment are shown above the box plots. mRNA includes BNT162b2 (BioNTech Pfizer) and mRNA-1273 (Moderna), and AZ refers to AstraZeneca ChAdOx1-S AZD1222. For simplicity, the HSCT groups in the plot include both Haematopoetic stem cell transplant and Chimeric Antigen Receptor T-cell (CAR-T) recipients. An SFC/10^6^ PBMCs of 4 or less was considered a negative response (represented by the dashed line).

*PBMC: peripheral blood mononuclear cells, SFC: spot-forming cells, V1: first vaccination, V2: second vaccination*

**
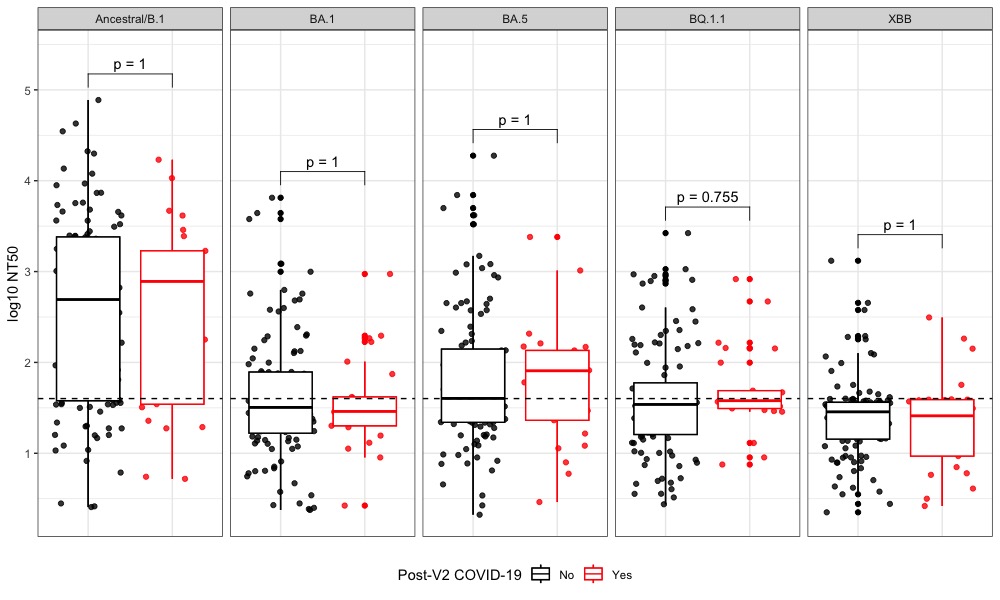
**

**Figure S4. Post-V2 NT50 values for HSCT/CAR-T recipients coloured by whether they had COVID-19 following their second vaccination, split by pseudotype tested**. Boxplots demonstrate the median and interquartile ranges (IQR25 and IQR75), and the whiskers the range. P values calculated using the Mann Whitney U test with Bonferroni adjustment are shown above the box plots.

*CAR-T: Chimeric Antigen Receptor T-cell, HSCT: Haematopoetic stem cell transplant, NT50: Half-maximal neutralising titre, V2: second vaccination*

*
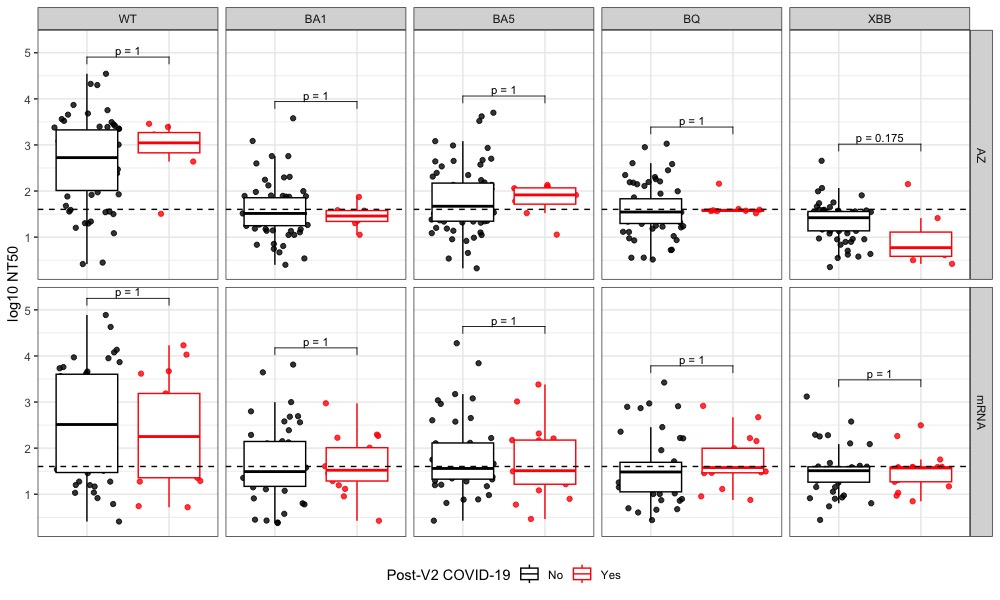
*

**Figure S5. Post-V2 NT50 values for HSCT/CAR-T recipients coloured by whether they had COVID-19 following their second vaccination. split by pseudotype and vaccination type received**. Boxplots demonstrate the median and interquartile ranges (IQR25 and IQR75), and the whiskers the range. P values calculated using the Mann Whitney U test with Bonferroni adjustment are shown above the box plots. mRNA includes BNT162b2 (BioNTech Pfizer) and mRNA-1273 (Moderna), and AZ refers to AstraZeneca ChAdOx1-S AZD1222.

*CAR-T: Chimeric Antigen Receptor T-cell, HSCT: Haematopoetic stem cell transplant, NT50: Half-maximal neutralising titre, V2: second vaccination*


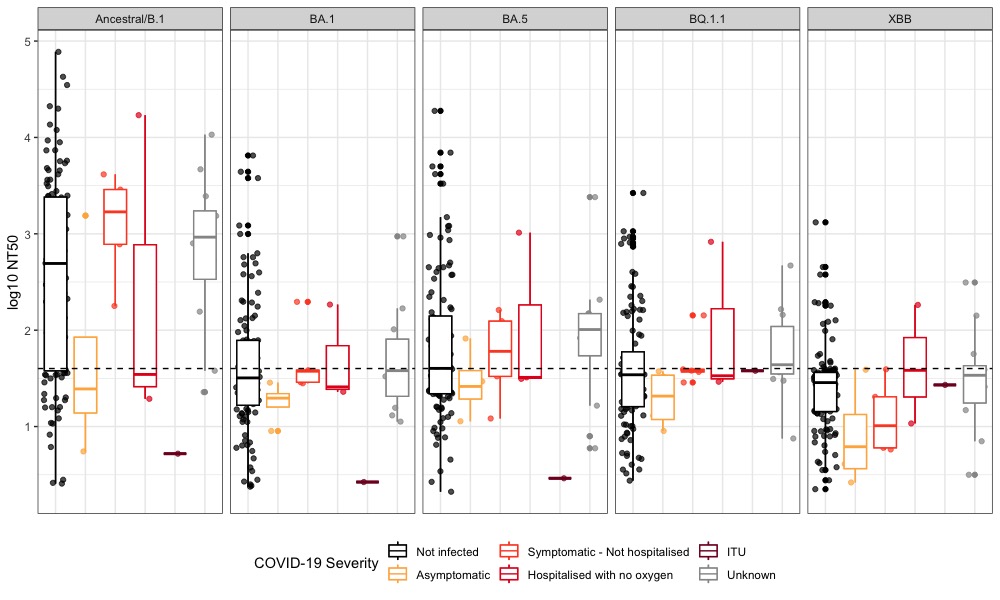


**Figure S6. Post-V2 NT50 values for HSCT/CAR-T recipients, split by severity of subsequent COVID-19 infection, split by pseudotype tested.** Boxplots demonstrate the median and interquartile ranges (IQR25 and IQR75), and the whiskers the range. P values (data not shown for simplicity of figure) were calculated using the Mann Whitney U test with Bonferroni adjustment and were not significant (p=1 for all groups).

*CAR-T: Chimeric Antigen Receptor T-cell, HSCT: Haematopoetic stem cell transplant, ITU: Intensive Treatment Unit, NT50: Half-maximal neutralising titre, V2: second vaccination*
