# Supplementary material for: Greater preservation of SARS‐CoV‐2 neutralising antibody responses following the ChAdOx1‐S (AZD1222) vaccine compared with mRNA vaccines in haematopoietic cell transplant recipients
Source: Br J Haematol. 2024 Nov 17;205(6):2206–18. doi: 10.1111/bjh.19874 (PMC11637739; doi:10.1111/bjh.19874)
Supplement: Supplementary file 2 — Appendix S2. [file BJH-205-2206-s002.docx]

# **OCTAVE Collaborative Group**

| **Affiliation** | **Name** |
| --- | --- |
| Patient and Public Representatives on the Trial Management Group | **Eilean MacDonald** |
|  | **Elspeth Insch** |
|  | **Holly Loughton** |
|  | **Richard Beesley** |
|  | **Vicky Churchill** |
| Cancer Centre, University Hospitals Birmingham NHS Foundation Trust, Birmingham. B15 2WB | **Andrew Filer** |
|  | **Gary Middleton** |
| Cancer Research UK Clinical Trials Unit (CRCTU), University of Birmingham, Birmingham B15 2TT UK | **Pam Kearns** |
|  | **Ana Hughes** |
|  | **Faye Lowe** |
|  | **Lucinda Billingham** |
|  | **Molly Harrison** |
|  | **Sarah Pirrie** |
|  | **Sophia Magwaro** |
| Cambridge University Hospitals NHS Foundation Trust, Addenbrooke’s Hospital, Department of Haematology, Cambridge CB2 OQQ UK | **Ram Malladi** |
|  | **Andrew King** |
|  | **Ben Uttenthal** |
|  | **Charles Crawley** |
|  | **Faisal Basheer** |
|  | **Sophie Lockey** |
|  | **Adetomilola Olaiya** |
|  | **Camilla Ward** |
|  | **Lee Mynott** |
| Imperial College London, Division of Digestive Diseases, Department of Metabolism, Digestion and Reproduction, Faculty of Medicine, London SW7 2AZ UK | **Benjamin H Mullish** |
|  | **Pinelopi Manousou** |
| Imperial College Healthcare NHS Trust, Hammersmith Hospital, Haematology Department, London W12 0HS UK | **Maria Atta** |
| Imperial College Healthcare NHS Trust, St Mary's Hospital, London W2 1NY UK | **Candice Clarke** |
|  | **Maria Prendecki** |
|  | **Paul Martin** |
|  | **Sarah Gleeson** |
|  | **Stephen McAdoo** |
|  | **Tina Thomson** |
| King's College Hospital NHS Foundation Trust, London, SE5 9RS UK | **Robin Sanderson** |
|  | **Daniele Avenoso** |
|  | **Victoria Potter** |
|  | **Pramila Krishmamurphy** |
|  | **Reuben Benjamin** |
|  | **Styliani Bouziana** |
|  | **Charlotte Graham** |
|  | **Varon Mehra** |
|  | **Prudence Hardefeldt** |
|  | **Maclaine Hipolito-Johnson** |
|  | **Angeline Pol** |
|  | **Adrienne Abioye** |
|  | **Christina Obiorah** |
| Leeds Institute of Medical Research, University of Leeds, Leeds LS2 9NL | **Claire Taylor** |
| Leeds Teaching Hospitals NHS Trust , Beckett Street, Leeds, West Yorkshire, LS9 7TF | **Karen Ingham** |
|  | **Stewart McConnell** |
|  | **Emma Binns** |
|  | **Lucy China** |
|  | **Abbie Javed** |
|  | **Frances Massey** |
| Northern Centre for Cancer Care, Freeman Hospital, Newcastle upon Tyne NE7 7DN UK | **Erin Hurst** |
|  | **Amy Publicover** |
|  | **Katy Scouse** |
|  | **Tom Fail** |
|  | **Tom Creasley** |
|  | **Matthew Collin** |
|  | **Caroline Shrubhsole** |
|  | **Kier Pickard** |
|  | **Susan Jones** |
|  | **Jessica Williams** |
|  | **Julie Twohig** |
|  | **Dianne Turner** |
| Oxford University Hospitals NHS Foundation Trust, John Radcliffe Hospital, Oxford, OX3 9DU UK | **Stavros Dimitiadis** |
|  | **Christine Sennett** |
|  | **Eleni Rountenko** |
| University of Oxford, Nuffield Department of Medicine, Oxford, OX1 2JD UK | **Thomas Marjot** |
|  | **Mr Nicholas M. Provine** |
|  | **Sophie L. Irwin** |
| Oxford University Hospitals NHS Foundation Trust, Churchill Hospital, Department of Oncology, Cancer and Haematology Centre, Oxford, OX3 7LE UK | **Murali Kesavan** |
|  | **Andy Peniket** |
|  | **Robert Danby** |
|  | **Ronjon Chakraverty** |
|  | **Nadeera Jayasekara** |
|  | **Sally Springett** |
|  | **Samiya Mahmood** |
|  | **Jacqueline Martin** |
| Sheffield Teaching Hospitals NHS Foundation Trust, Royal Hallamshire Hospital, Sheffield S10 2JF UK | **Naomi Meardon** |
|  | **Doreen Trown** |
| The University of Sheffield, Department of Infection, Immunity and Cardiovascular Disease, Sheffield S10 2TN UK | **Sam Hansford** |
|  | **Gurjinder Sandhar** |
| St George's Hospital and Medical School, St George’s University Hospitals NHS Foundation Trust, Department of Haematology, London SW17 0QT UK | **Mickey BC Koh** |
|  | **Patricia Faria** |
|  | **Paul Miller** |
| University of Birmingham, Institute of Immunology and Immunotherapy, Birmingham B15 2TT UK | **Paul Moss** |
|  | **Saly Al-Taei** |
| University Hospitals Birmingham NHS Foundation Trust, Department of Oncology, Birmingham B15 2GW UK | **Dan Rea** |
|  | **Hayley Rolfe** |
|  | **Tina McSkean** |
|  | **Alice Longe** |
|  | **Sarah Read** |
| University Hospitals Birmingham NHS Foundation Trust, National Institute for Health Research (NIHR) Birmingham Biomedical Research Centre, Centre for Liver and Gastrointestinal Research, Institute of Immunology and Immunotherapy, Birmingham B15 2TT UK | **Palak Trivedi** |
|  | **Diana Hull** |
| University Hospitals Birmingham NHS Foundation Trust, Department of Haematology, Birmingham B15 2TT UK | **Adrian Shields** |
|  | **Francesca Kinsella** |
|  | **Guy Pratt** |
|  | **Graham McIlroy** |
|  | **Helen Parry** |
| University of Glasgow, College of Medical, Veterinary & Life Sciences; Glasgow; G12 8QQ, UK | **Neil Basu** |
|  | **Andrew Melville** |
|  | **Ashley Gilmour** |
|  | **Aurelie Najm** |
|  | **Caron Paterson** |
|  | **Flavia Sunzini** |
|  | **James Brock** |
|  | **Matthew Rutherforfd** |
| University Hospital Southampton NHS Foundation Trust, Southampton General Hospital, Southampton SO16 6YD UK | **Chris Dalley** |
|  | **Deborah Richardson** |
|  | **Kate Hill** |
|  | **Mariam Amer** |
|  | **Clare Hutchison** |

# **OCTAVE-DUO Collaborative Group**

| **Affiliation** | **Name** |
| --- | --- |
| Patient and Public Representatives on the Trial Management Group with no affiliation | **Richard Beesley** |
|  | **Vicky Churchill** |
|  | **Elspeth Insch** |
|  | **Holly Loughton** |
|  | **Eilean MacDonald** |
| Imperial College London, Centre for Inflammatory Disease, Department of Immunology and Inflammation, Hammersmith Campus, Du Cane Road, London W12 0NN UK | **Maria Prendecki** |
|  | **Candic Clarke** |
|  | **Stacey McIntyre** |
|  | **Paige Mortimer** |
| Imperial College London, Dept of Immunology and Inflammation, London. UK | **Liz Lightstone** |
| Imperial College Healthcare NHS Trust, Hammersmith Hospital, London W12 OHS | **Thomas Walters** |
|  | **Sarah Gleeson** |
|  | **Paul Martin** |
|  | **Stephen McAdoo** |
| Imperial College London, Department of Infectious Diseases, School of Medicine, Chelsea and Westminster Hospital SW10 9NH UK | **Peter Kelleher** |
| King's College Hospital, London | **Robin Sanderson*** |
|  | **Jennifer Vidler** |
|  | **Donna Cassidy** |
|  | **Khristine Ordenes** |
|  | **Keith Senercz** |
|  | **Angeline Pol** |
|  | **Jessica Diedican** |
|  | **Benissa Narciso** |
| Leicester Royal Infirmary, Hope Clinical Trial Facility, Leicester. LE1 5WW, UK | **Matthew Ahearn*** |
|  | **Jayne Denyer** |
|  | **Rahima Ibrahim** |
| Royal Free Hospital, Clinical Immunology, Hampstead, London. NW3 2QG, UK | **Siobhan Burns*** |
|  | **Susan Tadros** |
|  | **Sarita Workman** |
| Royal Free Hospital, Hampstead, London. NW3 2QG, UK | **Janki Kavi** |
| Sheffield Teaching Hospitals NHS Foundation Trust, Royal Hallamshire Hospital, Sheffield S10 2JF UK | **Doreen Trown** |
| Sheffield Teaching Hospitals NHS Foundation Trust, Department of Haematology, Royal Hallamshire Hospital, Sheffield. S10 2JF, UK | **Rachael Selby** |
| The University of Sheffield, Department of Infection, Immunity and Cardiovascular Disease, The Medical School, Sheffield. S10 2RX, UK | **Naomi Meardon** |
| Leeds Teaching Hospitals NHS Trust , Beckett Street, Leeds, West Yorkshire, LS9 7TF | **Jennifer Clay** |
|  | **Karen Ingham** |
|  | **Helena Baker** |
|  | **Stewart McConnell** |
|  | **Emma Binns** |
|  | **Lucy China** |
|  | **Abbie Javed** |
|  | **Frances Massey** |
| Leeds Institute of Medical Research, University of Leeds, Leeds LS2 9NL | **Claire Taylor** |
| St George's Hospital, Cranmer Terrace, Tooting. SW17 0RE, UK | **Mickey Koh*** |
|  | **Julia Chackathayil** |
|  | **Patricia Faria** |
| University of Birmingham, Cancer Research UK Clinical Trials Unit (CRCTU), Edgbaston, Birmingham. B15 2TT, UK. | **Ana Hughes** |
|  | **Charlotte Gaskell** |
|  | **Dan Rea** |
|  | **Faye Lowe** |
|  | **Lucinda Billingham** |
|  | **Molly Harrison** |
|  | **Sarah Pirrie** |
|  | **Sophia Magwaro** |
|  | **Siân Lax** |
|  | **Biruk Asfaw** |
|  | **Faye Hatcliffe** |
|  | **Hayley Ellis** |
|  | **Martin Pope** |
|  | **Paula Wilshire** |
| University Hospitals Birmingham NHS Foundation Trust, Centre for Clinical Haematology, Birmingham B15 2SY, UK | **Helen Parry*** |
|  | **Francesca Kinsella** |
|  | **Hayley Rolfe** |
| University of Birmingham, Clinical Immunology Service, Edgbaston, Birmingham. B15 2TT, UK. | **Saly Al-Taei** |
| University College London Hospital, London. WC1E 6DD, UK | **Kwee Yong*** |
|  | **Kimberley Driver** |
|  | **Kaylee Gauntlett** |
|  | **Samir Ashek** |
|  | **Satyen Gohil** |
|  | **Tommy Rampling** |
|  | **William Townsend** |
|  | **Ke Xu** |
|  | **Sarah Guerrini** |
|  | **Marta Merida Morillas** |
|  | **Subarna Roy** |
|  | **Bhumika Patel** |
| University of Glasgow, College of Medical, Veterinary & Life Sciences, Glasgow. G12 8QQ, UK | **Maxine Arnott** |
|  | **Neil Basu** |
|  | **James Brock** |
|  | **Ashley Gilmour** |
|  | **Andrew Melville** |
|  | **Aurelie Najm** |
|  | **Matthew Rutherford** |
|  | **Flavia Sunzini** |
|  | **Andrew Farthing** |
|  | **Suzann Rundell** |
|  | **Emily Smith** |
|  | **Andrew Tong** |
|  | **Kieran Woolcock** |
| University of Oxford, Wellcome Centre for Human Genetics,Oxford, UK. | **Miles Carroll** |
|  | **Dung Nguyen** |
|  | **Tom Tipton** |
|  | **Stephanie Longet** |
|  | **Stephen Laidlaw** |
| University of Oxford, Nuffield Department of Medicine, Oxford, OX1 2JD, UK. | **Stavros Dimitriadis** |
|  | **Sophie Irwin** |
|  | **Paul Klenerman** |
|  | **Zixiang Lim** |
|  | **Thomas Marjot** |
|  | **Georgina Meacham** |
|  | **Sam M Murray** |
|  | **Nicholas Provine** |
|  | **Jack Satsangi** |
| Oxford University Hospitals NHS Trust, NIHR Oxford Biomedical Research Centre, Oxford, UK | **Paul Klenerman** |
| University of Oxford, Radcliffe Department of Medicine, Oxford. OX3 9DU, UK | **Ronjon Chakraverty** |
| University of Oxford, John Radcliffe Hospital, Oxford, OX1 2JD, UK | **Daniel Hanke** |
|  | **Zainab Malik** |
|  | **Victoria Walker** |
| University Hospital Southampton NHS Foundation Trust, Department of Haematology, Southampton. SO16 6YD, UK | **Clare Hutchison** |
|  | **Robert Lown** |
|  | **May N Lwin** |
| University Hospital Southampton NHS Foundation Trust, NIHR Southampton Clinical Research Facility, Southampton, SO16 6YD, UK | **Sarah Horswill** |
|  | **Nina Parungao** |
|  | **Stephen Saich** |
|  | **James Cullinane** |
| University Hospital Southampton NHS Foundation Trust, Department of Rheumatology, Southampton, SO16 6YD, UK | **Christopher Holroyd** |
| University Hospital Southampton NHS Foundation Trust, Southampton General Hospital, Tremona Road, Southampton. SO16 6YD, UK | **Gavin Babbage** |
| University of Southampton, Centre for Cancer Immunology, Southampton. SO16 6YD, UK | **Mark Gradwell** |
| COVID Surveillance Unit, Francis Crick Institute, London. NW1 1AT, UK | **Lou S Herman** |
|  | **Agnieszka Hobbs** |
|  | **Martina Ragno** |
|  | **Mary Y Wu** |
|  | **Murad Miah** |
|  | **Mauro Miranda** |
|  | **Nicola O’Reilly** |
|  | **Callie Smith** |
| British Society of Blood and Marrow Transplantation and Cellular Therapy, Guy's Hospital, London, SE1 9RT UK | **Paul Miller^** |
| UK Health Security Agency (UKHSA), Immunisation and Vaccine Preventable Diseases Division, UK Health Security Agency, London NW9 5EQ UK | **Georgina Ireland** |

* Principal Investigator at recruiting site.

^ Deceased.

**PROSECO Investigators**

| **Affiliation** | **Name** |
| --- | --- |
| Great Ormond Street Institute of Child Health Biomedical Research Centre, University College London, London, UK. | **Marina Johnson** |
| NIHR/Cancer Research UK Southampton Experimental Cancer Medicine Centre, WISH Laboratory, Southampton General Hospital, Southampton, UK. | **Adam R. Coleman**  **Adam Kelly**  **Andrew J. Davies** |
| Centre for Cancer Immunology, University of Southampton, Southampton, UK  Cancer Research UK Research Centre, University of Southampton, Southampton, UK. | **Anna H. Turaj** |
| Norfolk and Norwich University Hospitals NHS Foundation Trust, Norwich, UK. | **Victoria Willimott** |
| Department of Oncology, Bedford Hospital, Bedford, UK | **Anna Bowzyk Al-Naeeb** |
| Portsmouth Hospitals NHS Trust, Portsmouth, UK. | **Ann O’Callaghan** |
| Department of Clinical Haematology, Oxford University Hospitals NHS Foundation Trust, Oxford, UK. | **Graham P. Collins** |
| Department of Haematology, Newcastle upon Tyne Hospitals NHS Foundation Trust, Newcastle Upon Tyne, UK. | **Tobias Menne** |
| Nottingham University Hospitals NHS Trust, Nottingham, UK. | **Christopher P. Fox** |
| University Hospitals of Leicester NHS Trust, Leicester, UK. | **Matthew J. A’Hearne** |

**PITCH**

| **Affiliation** | **Name** |
| --- | --- |
| Oxford Vaccine Group, Department of Paediatrics, University of Oxford, Oxford, UK | **Teresa Lambe**  **Chris Dold**  **Sagida Bibi** |
| Translational and Clinical Research Institute, Newcastle University, Newcastle, UK. | **Christopher J A Duncan**  **Rebecca Payne** |
| Nuffield Dept of Clinical Neurosciences, University of Oxford, UK. | **Donal Skelly** |
| HPRU in Emerging and Zoonotic  Infections Institute of Infection, Veterinary and Ecological Sciences, University of Liverpool,  Liverpool, UK. | **Daniel G Wootton**  **Shona C Moore**  **Lance Turtle**  **Sue L Dobson** |
| Immunisation and Vaccine Preventable Diseases Division, UK Health Security Agency, London NW9 5EQ, UK. | **Victoria Hall**  **Susan Hopkins**  **Jasmin Islam**  **Ashley Otter**  **Sarah Foulkes** |
| MRC Toxicology Unit, University of Cambridge, Cambridge,  CB1 1QR, UK. | **James E D Thaventhir** |
| Institute for Immunology and Immunotherapy, College of Medical and Dental Science, University of Birmingham, Birmingham, B15 2TT, UK | **Sian Faustini** |
| Nuffield Department of Medicine, University of Oxford, Oxford, UK | **Alexandra Deeks**  **Anni Jamsen**  **Anthony Brown**  **Chris Conlon**  **Barbara Kronsteiner**  **Priyanka Abraham**  **Eloise Phillips**  **Alexander Hargreaves**  **Lisa Frending**  **Lizzie Stafford**  **Mohammad Ali**  **Patpong Rongkard**  **Alex Mentzer**  **Eleanor Barnes**  **Susanna J Dunachie**  **Paul Klenerman**  **Sandra Adele**  **Simon Travis**  **Siobhan Gardiner**  **Miles Carroll**  **John Frater**  **Stephanie Longet**  **Tom Malone** |
| Clinical Immunology Service, University of Birmingham, Edgbaston, Birmingham, UK | **Alex G Richter** |
| Oxford University Hospitals NHS Foundation Trust, Oxford, UK | **Katie Jeffery**  **Eleanor Barnes**  **Susanna J Dunachie** |
| Division of Clinical Medicine, University of Sheffield, Sheffield, UK | **Sarah L Rowland-Jones**  **Thushan de Silva** |
